# A Novel Approach for the Discovery of Biomarkers of Radiotherapy Response in Breast Cancer

**DOI:** 10.3390/jpm11080796

**Published:** 2021-08-14

**Authors:** James Meehan, Mark Gray, Carlos Martínez-Pérez, Charlene Kay, Jimi C. Wills, Ian H. Kunkler, J. Michael Dixon, Arran K. Turnbull

**Affiliations:** 1 Translational Oncology Research Group, Institute of Genetics and Cancer, Western General Hospital, University of Edinburgh, Edinburgh EH4 2XU, UK; carlos.martinez-perez@ed.ac.uk (C.M.-P.); charlene.kay@ed.ac.uk (C.K.); a.turnbull@ed.ac.uk (A.K.T.); 2 The Royal (Dick) School of Veterinary Studies and Roslin Institute, University of Edinburgh, Midlothian EH25 9RG, UK; mark.gray@ed.ac.uk; 3 Breast Cancer Now Edinburgh Research Team, Institute of Genetics and Cancer, Western General Hospital, University of Edinburgh, Edinburgh EH4 2XU, UK; mike.dixon@ed.ac.uk; 4 Cancer Research UK Edinburgh Centre, Institute of Genetics and Cancer, University of Edinburgh, Edinburgh EH4 2XU, UK; jimi@firefinch.io (J.C.W.); iankunkler@yahoo.com (I.H.K.); 5 Firefinch Software Ltd., Edinburgh EH12 9DQ, UK

**Keywords:** breast cancer, radiotherapy, radiosensitivity biomarkers, secretome, radioresistance

## Abstract

Radiotherapy (RT) is an important treatment modality for the local control of breast cancer (BC). Unfortunately, not all patients that receive RT will obtain a therapeutic benefit, as cancer cells that either possess intrinsic radioresistance or develop resistance during treatment can reduce its efficacy. For RT treatment regimens to become personalised, there is a need to identify biomarkers that can predict and/or monitor a tumour’s response to radiation. Here we describe a novel method to identify such biomarkers. Liquid chromatography-mass spectrometry (LC-MS) was used on conditioned media (CM) samples from a radiosensitive oestrogen receptor positive (ER^+^) BC cell line (MCF-7) to identify cancer-secreted biomarkers which reflected a response to radiation. A total of 33 radiation-induced secreted proteins that had higher (up to 12-fold) secretion levels at 24 h post-2 Gy radiation were identified. Secretomic results were combined with whole-transcriptome gene expression experiments, using both radiosensitive and radioresistant cells, to identify a signature related to intrinsic radiosensitivity. Gene expression analysis assessing the levels of the 33 proteins showed that 5 (YBX3, EIF4EBP2, DKK1, GNPNAT1 and TK1) had higher expression levels in the radiosensitive cells compared to their radioresistant derivatives; 3 of these proteins (DKK1, GNPNAT1 and TK1) underwent in-lab and initial clinical validation. Western blot analysis using CM samples from cell lines confirmed a significant increase in the release of each candidate biomarker from radiosensitive cells 24 h after treatment with a 2 Gy dose of radiation; no significant increase in secretion was observed in the radioresistant cells after radiation. Immunohistochemistry showed that higher intracellular protein levels of the biomarkers were associated with greater radiosensitivity. Intracellular levels were further assessed in pre-treatment biopsy tissues from patients diagnosed with ER^+^ BC that were subsequently treated with breast-conserving surgery and RT. High DKK1 and GNPNAT1 intracellular levels were associated with significantly increased recurrence-free survival times, indicating that these two candidate biomarkers have the potential to predict sensitivity to RT. We suggest that the methods highlighted in this study could be utilised for the identification of biomarkers that may have a potential clinical role in personalising and optimising RT dosing regimens, whilst limiting the administration of RT to patients who are unlikely to benefit.

## 1. Introduction

Radiotherapy (RT), initially utilised for cancer treatment in the 1890s [1], still has a crucial role in the multidisciplinary management of breast cancer (BC) today, in spite of many advances in both surgery and systemic therapy. Studies have shown that RT can benefit up to 83% of BC patients [2] and that whole-breast RT following breast-conserving surgery provides local control and survival rates comparable to mastectomy [3,4,5]. Unfortunately, not all BC patients obtain a therapeutic benefit from RT; although overall five-year BC survival rates after RT are ~80%, it has been estimated that local recurrences or metastatic disease will develop in 30% of these patients, the majority of whom will die within 5 years [6]. In BC and other solid tumours, the clinical effects of RT are also only observed near the end or after the treatment course has been completed; as such, patients who do not respond to RT (due to either innate [7] or acquired radioresistance [8]), will initially go undetected. This delay in identifying non-responding cancers exposes patients to the risk of acquiring RT-induced side effects for no therapeutic gain [9], allows tumour progression, impacts long-term survival and delays the delivery of alternate, more effective treatments [10].

The precision medicine initiative is a concept that is increasingly being implemented into BC clinical practices. It can be defined as the prevention, examination and treatment of disease, while also considering individual variability [11]. Molecular classification systems, based on gene expression signatures of BC tissue, are currently being used to classify these cancers into specific subtypes that can predict prognosis and treatment response [12,13,14,15,16]. While these tools have led to improvements in the systemic treatment of BC patients, the incorporation of RT into the precision medicine initiative is lagging behind such achievements [17]. To improve BC patient outcomes and allow RT to become fully integrated into the precision medicine initiative, we need to identify biomarkers that can not only predict RT response before the initiation of treatment but also allow the evaluation of a tumour’s response to RT during treatment [18]. These biomarkers could enable personalised RT treatment regimens to be given to individuals on the basis of individual risk and tumour biology and also allow the identification of patients who are unlikely to benefit from RT.

In response to this unmet clinical need, studies have attempted to produce radiation sensitivity gene signatures that can predict tumour radiation response and identify those resistant to conventional RT regimens [19,20,21,22]. Unfortunately, as of yet, none of these gene signatures have been sufficiently validated for clinical use. Rather than using tissue-based biomarkers, another method that could be used to personalise RT is the detection and/or measurement of tumour secreted biomarkers. Several secretomic studies have used conditioned media (CM, spent media harvested from cultured cells) from BC cell lines cultured in vitro in an attempt to detect clinically relevant biomarkers [23,24,25,26,27]. While these secretomic studies have distinguished novel biomarkers of aggressive phenotypes [23,25] or biomarkers that act as predictors of chemotherapy response [26], no study has yet explored the immediate impact of radiation on the secretome of cancer cells as a means of evaluating radiation response and/or determining radiosensitivity [28]. 

**Figure 1:**
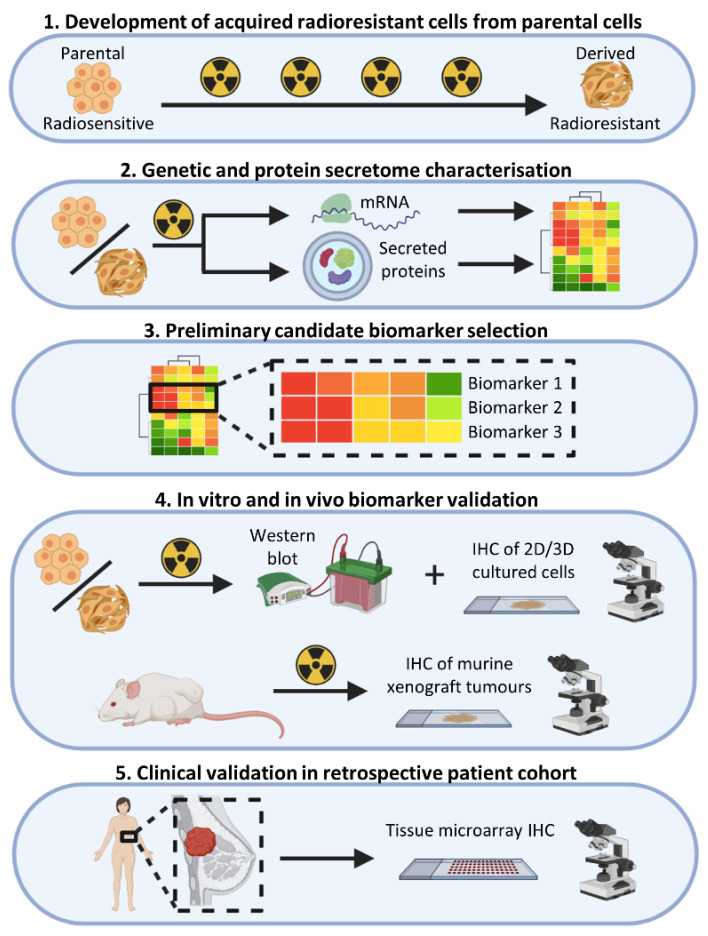
Biomarker discovery pipeline. Outline of the methods used to identify and validate biomarkers of BC RT response. Figure created with Biorender.com.

We have previously developed and characterised radioresistant (RR) cells derived from oestrogen receptor positive (ER^+^) BC cell lines [29]. In-depth genotypic, phenotypic and functional characterisation identified several important mechanisms (including EMT, reduced proliferation, metabolic changes and activation of PI3K, AKT and WNT signalling) that may contribute to the development of radioresistance. In this current study, we utilised these RR models, along with their parental cells, to describe a novel method for the identification of gene, intracellular protein and secreted protein biomarkers that can be used to provide prognostic and/or predictive information on a tumour’s response to RT. Utilising secretomic data obtained through liquid chromatography-mass spectrometry (LC-MS) with a radiosensitive ER^+^ BC cell line (MCF-7), we characterised the cancer secretome and identified cancer-secreted biomarkers whose release reflected an acute radiation response. In addition, we combined the secretomic results with data from whole-transcriptome gene expression experiments, using both radiosensitive and resistant cells, to identify a signature related to intrinsic radiosensitivity. Candidate secreted and intracellular biomarkers were then successfully validated in-lab using cell lines, BC xenograft tumours and patient tissue samples (Figure 1). We suggest that our methods can be utilised for the identification of biomarkers that could have a clinical role in personalising RT dosing regimens, thus optimising treatment and limiting the administration of RT to patients who are unlikely to benefit.

## 2. Materials and Methods

### 2.1. Cell Culture

Unless indicated otherwise, cell culture reagents were acquired from Gibco Thermo Fisher Scientific (Loughborough, England). MCF-7 and ZR-751 BC cell lines were cultured in Dulbecco’s Modified Eagle’s Medium (DMEM) supplemented with 10% foetal calf serum, 50 U mL^−1^ penicillin and 50 mg mL^−1^ streptomycin. Cells were incubated at 37 °C in a humidified atmosphere with 5% CO_2_. These cell lines were obtained from the American Type Culture Collection (LGC Standards, Teddington, England). Cells were authenticated by short tandem repeat profiling carried out at Public Health England (Porton Down, Salisbury, England). Spinner flasks (Cellcontrol Spinner Flask, Integra, Zizers, Switzerland), placed onto a magnetic stirrer platform (Cellspin, Integra, Zizers, Switzerland), were used to produce multicellular tumour spheroids (MTS) from single cell suspensions. MTS were allowed to form over 7 days in normal incubation conditions before use.

### 2.2. Irradiation of Cells and Development of Radioresistant Cell Lines

Radioresistant (MCF-7 RR and ZR-751 RR) cells were established from their parental cell lines within our lab, as described previously [29]. Briefly, parental cell lines were treated with weekly doses of radiation using a Faxitron cabinet X-ray system 43855D (Faxitron X-ray Corporation, Lincolnshire, IL, USA). After a starting dose of 2 Gy, the radiation doses were increased by 0.5 Gy per week over a three-month period. Cells were subsequently maintained with additional weekly doses of 5 Gy after the development of radioresistance.

### 2.3. Cell Irradiation and Secretome Sample Preparation

Cells were seeded into six well plates to achieve ~40–50% confluency at 24 h. Cells were washed three times with PBS before 2 mL of serum-free media (SFM) was added. The cells were serum-starved for 2 h. Cells were then exposed to radiation and the CM was harvested at appropriate time points. Secretome samples underwent processing for LC-MS or western blot (WB) analysis immediately following collection. Following CM harvesting, cells were routinely trypsinised and counted using a haemocytometer with trypan blue exclusion (Sigma-Aldrich, Gillingham, England). 

CM samples were centrifuged at 3000× *g* for 15 min at 4 °C to remove dead cells and large debris. Proteins were concentrated from the supernatant using the Amicon Ultra-0.5 Centrifugal Filter Unit with Ultracel-3 membrane (Merck Millipore, Livingston, Scotland) as per the manufacturer’s protocol. Briefly, 500 µL of the CM was added to the Amicon Ultra filter device and the sample was centrifuged at 14,000× *g* for 30 min at 4 °C. The filter was removed and placed upside down into a new 1.5 mL microcentrifuge tube. The sample was centrifuged at 1000× *g* for 2 min at 4 °C to elute the concentrated protein. The ultrafiltrate was then stored at −80 °C.

### 2.4. Liquid Chromatography-Mass Spectrometry and Secretome Analysis

In-solution digests of secretomic samples were performed for LC-MS analysis. Protein concentrations of the CM samples were ascertained using a bicinchoninic acid assay (Sigma-Aldrich, Gillingham, England). 50 µg of protein was added to 100 mM tris/2 M urea/10 mM DTT and heated for 30 min at 50 °C; this was performed in 96 well plates with silicon lids. 55 mM iodoacetamide was then added and incubated in darkness for 30 min at room temperature. After this, trypsin (1:100 dilution) was added and incubation was performed overnight at room temperature. Of this peptide solution, 10 µg was inserted into an activated (20 µL methanol) and equilibrated (100 µL 0.1% trifluoroacetic acid (TFA)) C18 StAGE tip; washing was performed with 100 µL of 0.1% TFA. The bound peptides were eluted into Protein LoBind tubes with 20 µL of 80% acetonitrile (ACN) and 0.1% TFA solution. The samples were concentrated to volumes <4 µL using a vacuum concentrator. Final sample volumes were adjusted to 6 µL using 0.1% TFA. Online LC was performed using a Dionex RSLC Nano. After the C18 clean-up, 5 µg of the peptide solution was injected onto a C18 packed emitter and eluted over a gradient of 2–80% ACN for 2 h with 0.1% TFA. Eluted peptides were ionised at +2 kV and data-dependent analysis was carried out on a Thermo Q-Exactive Plus. MS1 was obtained with resolution 70,000 and mz range 300–1650 and the top 12 ions were chosen for fragmentation with a normalised collision energy of 26 and an exclusion window of 30 sec. MS2 was collected with a resolution of 17,500. The AGC targets for MS1 and MS2 were 3 × 10^6^ and 5 × 10^4^, respectively. All spectra were obtained with 1 microscan without lockmass.

Data were analysed using MaxQuant in conjunction with uniport fasta database with matching between runs. Prior to the analysis, all data were log_2_ transformed. For fold change analysis, data were normalised to untreated controls at each time point using R (Bioconductor) software and packages [30]. Venn diagrams were generated using jvenn [31]. Heatmap and cluster analyses were performed using TM4 MeV (multiple experiment viewer) software [32]. Heatmap clustering was carried out using Pearson correlation with average linkage. Protein interaction networks of candidate biomarkers were generated using the STRING protein interaction database [33] and Markov clustering algorithms [34]. All secretomic datasets generated and/or analysed within this study are available on the PRoteomics IDEntifications Database (PRIDE) [35,36]; these can be found with the PRIDE project accession number PXD027572.

### 2.5. Lactate Dehydrogenase Assay

Lactate dehydrogenase (LDH) levels within the CM used for secretome analysis were analysed to confirm the absence of cell death after radiation treatment. LDH levels were measured using the CyQUANT LDH Cytotoxicity Assay Kit (Invitrogen, Inchinnan, Scotland) as per the manufacturer’s protocol. Briefly, 50 µL of CM was transferred to a 96-well plate, along with 50 µL of the reaction mixture. The plates were incubated at room temperature for 30 min. 50 µL of stop solution was then added to the wells and absorbance was measured at 490 nm and 680 nm using a Spark 20M multimode reader (Tecan, Männedorf, Switzerland).

### 2.6. RNA Extraction and Whole-Transcriptome Gene Expression Analysis

Cells were seeded into 75 mm plates (3 × 10^6^ cells/plate). Following 24 h of incubation, cells were serum-starved for 2 h (providing the same experimental conditions as for CM collection) and then exposed to radiation. Pellets containing up to 10,000,000 cells were collected by trypsinisation at 0, 2 and 8 h post-radiation, snap-frozen on dry ice and stored at −70 °C. RNA was extracted from the cells with the RNeasy Mini Kit using QIAshredder technology (Qiagen, Manchester, England). Spin technology was used to purify total RNA from the cells, as per the manufacturer’s protocol. RNA was quantified and examined for contaminants using the NanoDrop^TM^ Spectrophotometer ND1000 and the Qubit RNA IQ Assay (Thermo Fischer Scientific, Loughborough, England). RNA quality was assessed by producing RNA integrity numbers (RIN) for each of the samples using the Agilent Bioanalyzer (Agilent Technologies Ltd., Stockport, England); each sample had RIN values above 9.7 (Supplementary Table S1). The ZR-751 2 h 2 Gy sample failed in sequencing and was removed from further analysis. Lexogen QuantSeq 3′ FWD sequencing technology produced full genome expression read-counts on an Illumina flow cell; these were scanned using the Illumina HiScanSQ system (Edinburgh Clinical Research Facility, University of Edinburgh, Scotland). Next-generation sequencing reads were generated towards the poly(A) tail with read 1 directly reflecting the mRNA sequence. The FASTQ files were pre-processed with the BlueBee high-performance next generation sequencing analysis software; this uses poly(A) tail trimming and alignment to the Genome Reference Consortium Human genome build 38 reference genome using the Spliced Transcripts Alignment to a Reference (STAR) algorithm [37]. 

Filtering was carried out on the data, removing all genes that had fewer than five reads per sample in at least 90% of samples. Overall, 17,243 genes were mapped to human Ensembl gene identifiers. Data were log_2_ transformed and quantile normalised in R (Bioconductor) software and packages [30] before any analysis was carried out. Heatmap and cluster analyses were performed with the TM4 MeV (multiple experiment viewer) software [32]. Heatmap clustering was implemented using Pearson correlation with average linkage. Correction for batch effects was performed to integrate gene expression data produced in this study with public datasets; this was carried out using the ComBat package in R, as described previously [38,39]. Gene enrichment analysis was performed in DAVID Functional Annotation Bioinformatics Microarray Analysis [40] and also using the KEGG [41] and Reactome [42,43] databases. Differential gene expression analysis was performed using ranked products with a false discovery rate of 0.01. All gene transcriptomic datasets generated and/or analysed within this study are available in the NCBI’s Gene Expression Omnibus [44]; these can be found with the GEO Series accession number GSE120798.

### 2.7. Protein Isolation and Detection

Whole-cell lysates were procured as previously described [45], with protein concentrations ascertained using a bicinchoninic acid assay. Sodium dodecyl sulphate (SDS) polyacrylamide gel electrophoresis was used to separate proteins. After separation, proteins were transferred to Immobilon-P transfer membranes (Merck Millipore, Livingston, Scotland). Membranes were incubated in LI-COR Odyssey blocking buffer solution (1:1 with PBS) for 1 h at room temperature. The membranes were then incubated overnight at 4 °C with primary antibodies DKK1 (abcam ab93017), GNPNAT1 (abcam ab234981) and TK1 (abcam ab76495). IRDye 800CW and IRDye 680LT fluorescently labelled secondary antibodies (LI-COR, Bioscience, Cambridge, England), diluted in LI-COR Odyssey blocking buffer solution, were used to bind to the primary antibodies. An LI-COR Odyssey Imager was used to detect the presence of signals from the bound secondary antibodies. 

### 2.8. Murine Xenograft Experiments

As part of a complementary study, radiation-treated mouse xenograft tissue was available for analysis. These in vivo murine studies were undertaken under a UK Home Office Project Licence in accordance with the Animals (Scientific Procedures) Act 1986. All experiments received approval from the University of Edinburgh Animal Welfare and Ethical Review Board. The recommended guidelines for the welfare and use of animals in research were followed. CD-1 immunodeficient female nude mice (Charles River Laboratories, Tranent, Scotland) ≥8 weeks of age were allowed at least a seven-day period of acclimatisation to a sterile, pathogen-free environment with ad libitum access to food and water. Mice were housed in groups of five in individually ventilated cages in a barrier environment. 

Approximately 5 × 10^8^ MCF-7 and MCF-7 RR cells were grown routinely and re-suspended in individual aliquots of 0.5 mL of SFM and 0.5 mL of Matrigel Matrix (Corning, Ewloe, Wales). Under gaseous isoflurane anaesthesia, each mouse received a 0.72 mg 17B-Oestradiol pellet (60-day release, Innovative Research of America, Sarasota, FL, USA) implanted subcutaneously in the dorsum using a 10 G trocar. 0.1 mL of either the MCF-7 or MCF-7 RR cell suspension was injected bilaterally into subcutaneous flank tissue using a 22 G needle connected to a 1 mL syringe. Once stock tumours had grown to ~1.0 cm in length mice were euthanised by cervical dislocation. In a sterile cabinet, xenograft tumours were harvested and placed into DMEM with no additives and sectioned into fragments ~1–2 mm in length. Implantation of tumour fragments into experimental mice was performed under gaseous isoflurane anaesthesia using a 12 G trocar. Each mouse received a 0.72 mg 17B-Oestradiol pellet as previously described and one tumour fragment was injected into the subcutaneous tissue of the flank. Mice were monitored for the development of xenograft tumours which occurred within 6–8 weeks post-implantation. Once the tumours had grown to ~1.0 cm in length, they were radiated. Mice were euthanised 24 h post-radiation and the tumours harvested (*n* = 5). Control tumours were left untreated and harvested at the same time (*n* = 5). 

### 2.9. Human Breast Tissue Experiments

To investigate whether candidate biomarkers could predict response to RT we identified ER^+^ positive breast cancer patients within a unique series of patient-derived BC tissues known as the Edinburgh Breast-Conserving Series (BCS) [46]. The Edinburgh BCS comprises a fully documented consecutive cohort of 1812 patients treated by breast conservation surgery, axillary node sampling or clearance and whole breast radiotherapy between 1981 and 1998. Over the study period, patients were managed by a specialist multidisciplinary team of surgeons, radiologists, pathologists and oncologists. Patients were those considered suitable for breast-conserving therapy and were T1 or T2 (<30 mm), N0 or N1 and M0 based on conventional TNM staging. Post-operative breast radiotherapy was given over 4–5 weeks at a dose of 45 Gy in 20–25 fractions. Notably, 12.7% of patients received no additional adjuvant therapy (chemotherapy or endocrine therapy) and of those 37% were ER-rich tumours (*n* = 80). It is these cases which were selected for analysis in this study. Clinicopathological data were available, including patient age, lymph node status, ER and PR status, tumour size and grade (see Supplementary Table S2). To generate tissue microarrays (TMAs) from these patients, formalin-fixed paraffin-embedded tissue blocks were initially created from patient-derived surgical excision specimens. These blocks were analysed by a pathologist to identify tumour regions. TMAs were then constructed in triplicate with representative cores (diameter ~700 μm) taken from three different random areas of the tumour. Each of the triplicates was then placed into three different TMA blocks. For use in our study, these three blocks were stained independently to assess intracellular protein levels of candidate biomarkers. The staining results of the three matched cores were then averaged. Following TMA processing, between 74 and 78 cases with intact triplicate samples were available for analysis. Recurrence-free survival data were available with a median follow-up of 12.7 years. Ethical approval for the study was granted under the Lothian NRS BioResource approval number 20/ES/0061. 

### 2.10. Immunohistochemistry

Immunohistochemistry (IHC) was performed on formalin-fixed human TMAs, MTS and murine xenograft tumours, in addition to methanol-fixed cells cultured in Lab-Tek II chamber slides (Thermo Fisher Scientific, Loughborough, England). Formalin-fixed samples were deparaffinised and rehydrated, after which antigen retrieval was performed. 3% H_2_O_2_ (Dako, Ely, England) was used to block endogenous peroxidase activity. All samples were incubated with Total Protein Block (Dako, Ely, England) for 1 h at room temperature. Primary antibodies DKK1 (abcam ab93017), GNPNAT1 (abcam ab234981) and TK1 (abcam ab76495) were incubated for 1 h at room temperature. One drop of Envision labelled polymer (Dako, Ely, England) was added to each sample for 30 min, after which DAB and substrate buffer (Dako, Ely, England) was applied for 10 min. Haematoxylin was used to counterstain the tissues, after which the slides were dehydrated and mounted with coverslips using a DXP mountant (Sigma-Aldrich, Gillingham, England).

IHC scoring of the Breast-Conserving Series TMAs was performed independently by two researchers. The scoring system used depended on the staining pattern observed. If staining intensity was consistent within a sample for a candidate biomarker (DKK1 and GNPNAT1), the scores given ranged from 0 (no staining), 1+ (weak staining), 2+ (moderate staining) and 3+ (strong staining). If staining intensity varied within a sample (TK1), then each sample was given a score that was dependent on the staining intensity (0, 1+, 2+ or 3+) combined with the percentage of cells with that intensity of staining, providing a final score ranging from 0–300. 

### 2.11. Statistical Analysis

One-way ANOVA, with Holm–Šídák multiple comparisons tests, was used to check for differences in secretion levels of candidate biomarkers within a cell line in the western blot CM experiments. Two-way ANOVA tests were performed to assess for differences in intracellular levels of candidate biomarkers between parental and RR cell lines in the western blot experiments using whole-cell lysate samples. For the Kaplan–Meier analysis of recurrence-free survival data in relation to candidate biomarker expression levels, the *p*-value was derived from log-rank (Mantel-cox) tests. The *p*-values ≤ 0.05 were deemed statistically significant. Graphs and statistical analysis were generated with GraphPad Prism 9 for Windows (GraphPad Software Ltd., San Diego, CA, USA).

## 3. Results

### 3.1. Characterisation of the MCF-7 Basal Secretome

**Figure 2:**
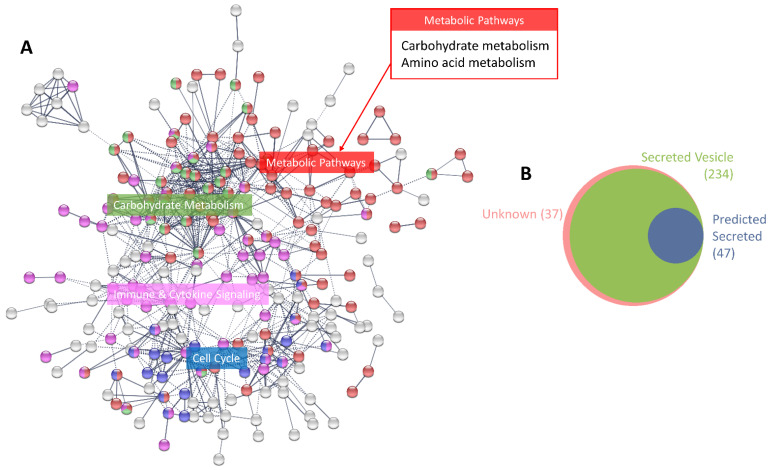
Characterisation of the MCF-7 basal secretome. (**A**) Functional protein association network showing the subset of 231 secreted proteins with known interactions from the total 318 proteins identified in the untreated basal secretome (after cut-offs were applied). Graph produced in STRING based on co-expression with high-confidence interaction score (0.7), clustered using the Markov Clustering algorithm. Significantly enriched pathways from the KEGG [41] and Reactome [42,43] databases are highlighted and labelled (lists of proteins in each pathway are provided in Supplementary Table S3). (**B**) Venn diagram showing proportions of proteins identified in the basal secretome and their reported/predicted method of secretion; (pink) unknown, (green) secreted in exosomes/microvesicles (ExoCarta [47] and Vesiclepedia [48]), and (blue) directly secreted (Human Protein Atlas [49], SignalP [50], Phobius [51] and SPOCTOPUS [52]).

Initial analysis was performed using CM samples procured from untreated MCF-7 cells 24 h after serum starvation to characterise the basal secretome before irradiation. The total number of proteins identified in the untreated secretome was 808. A cut-off of 2 was used to enable a functional analysis to be performed for the identification of key enriched pathways. Using this cut-off value, 318 proteins were detected within the CM of untreated MCF-7 cells; of these, 231 were shown to interact with one another. These secreted proteins were predominately involved in metabolic pathways, immune and cytokine signalling and cell cycle regulation (Figure 2A). The majority of these proteins have been reported/predicted to be secreted in exosomes/microvesicles or are released directly; only 37 had an unknown method of secretion (Figure 2B).

### 3.2. Characterisation of the MCF-7 Radiation-Induced Secretome

Following characterisation of the MCF-7 untreated secretome, we wished to identify differentially secreted proteins in response to radiation. To achieve this, MCF-7 cells were treated with a single dose of 2 Gy and CM samples were obtained up to 24 h post-radiation. To ensure that radiation treatment was not causing significant cell death, cell counts (using trypan blue exclusion) and LDH quantification (using CM from these cells) were performed. Results demonstrated no difference in total cell numbers or LDH levels between untreated and radiation treated groups at 24 h (Supplementary Figure S1). 

**Figure 3:**
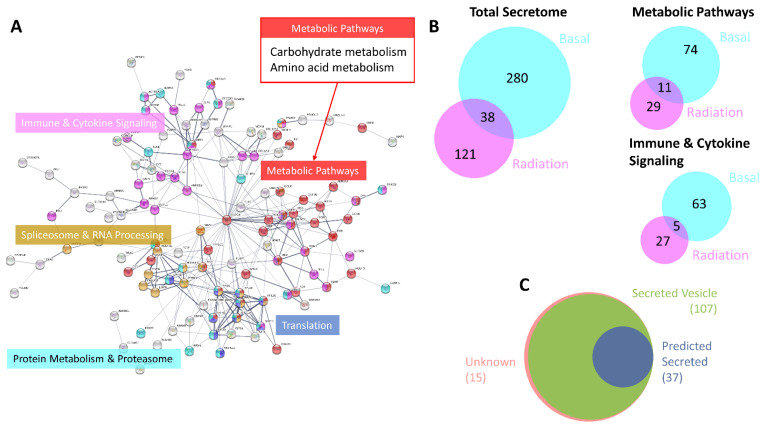
Characterisation of radiation-induced MCF-7 secretome. (**A**) Functional protein association network of the subset of 120 proteins with known interactions from the total number of 159 secreted proteins at 24 h with at least a 50% increase in secretion level following 2 Gy of radiation compared with 24 h untreated controls. Graph produced in STRING based on co-expression and reported STRING interactions with high-confidence interaction score (0.7), clustered using the Markov Clustering algorithm. Significantly enriched pathways from the KEGG [41] and Reactome [42,43] databases are highlighted and labelled (lists of proteins in each pathway are provided in Supplementary Table S4). (**B**) Venn diagrams showing the overlap in secreted proteins between the basal secretome and the radiation-induced secretome in respect of all secreted proteins and enriched pathways in both secretome profiles. (**C**) Venn diagram showing proportions of proteins identified in the radiation-induced secretome and their reported/predicted method of secretion; (pink) unknown, (green) secreted in exosomes/microvesicles (ExoCarta [47] and Vesiclepedia [48]), and (blue) directly secreted (Human Protein Atlas [49], SignalP [50], Phobius [51] and SPOCTOPUS [52]).

The total number of proteins detected in the CM 24 h after 2 Gy was 552. A total of 159 proteins were identified which exhibited at least a 50% increase in secretion levels following 2 Gy of radiation compared with 24 h untreated controls. As in the basal secretome, some of the secreted proteins were involved in immune and cytokine signalling and metabolism, whereas proteins involved in translation, spliceosome, RNA processing, protein metabolism and the proteasome were found only in the secretome of irradiated MCF-7 cells (Figure 3A). While there was some overlap between the secretomes of untreated and treated cells, the majority of the proteins isolated in the irradiated secretome were not found in the basal CM (Figure 3B). Like the basal secretome, most of the proteins identified in the radiation secretome were reported/predicted to be secreted (Figure 3C).

**Figure 4:**
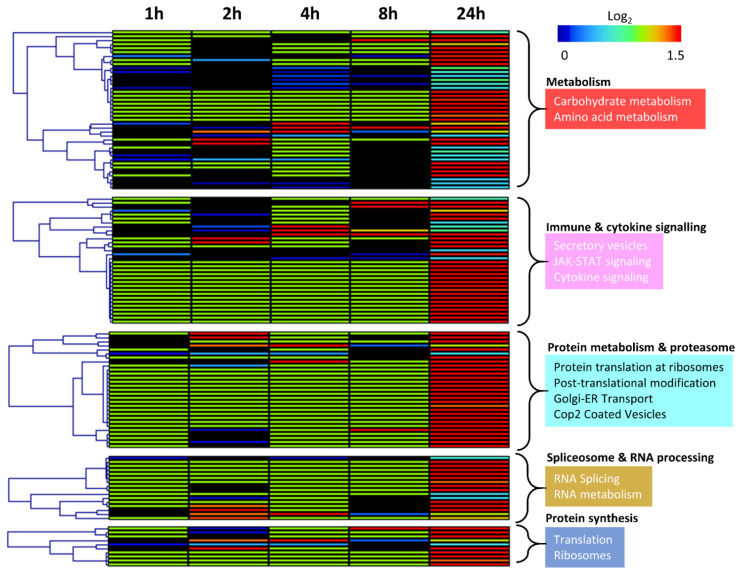
Comparison of secreted protein level by enriched pathways across all timepoints. Heatmap is based on log_2_ secretion levels following 2 Gy of radiation at 1, 2, 4, 8 and 24 h compared to untreated controls at each timepoint in respect of pathways enriched in the radiation-induced secretome. Functional enrichment was performed in STRING using the KEGG [41] and Reactome [42,43] databases. Clustering of proteins is based on Pearson correlation with average linkage. Heatmap colours denote log_2_ change in secretion level compared to untreated controls at each time point as denoted by the colour bar.

Analysis was performed to assess differences in the enriched pathways identified in the 24 h treated secretome across earlier time points. Secretion levels, relative to untreated controls at each time point, were assessed following 2 Gy of radiation at 1, 2, 4, 8 and 24 h (Figure 4). Results showed that the pathways enriched in the secretome at 24 h were also identified at the earlier time points, but secretion levels of the proteins were highest at 24 h. These results provided justification for focusing on the 24 h time point for biomarker discovery.

### 3.3. Gene Expression Changes Associated with Response to Radiation in Parental Radiosensitive and Derived Radioresistant MCF-7 Cells

**Figure 5:**
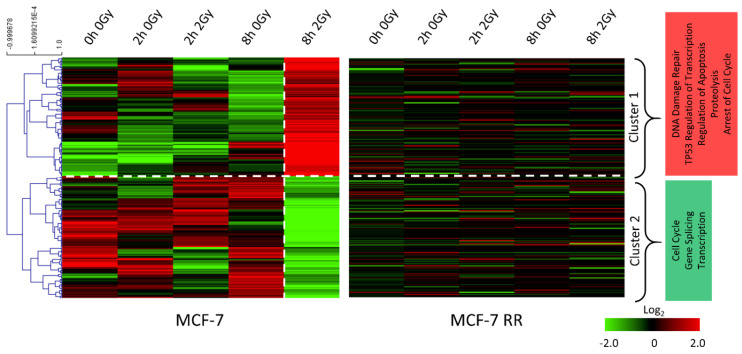
MCF7 and MCF-7 RR gene expression changes associated with response to radiation. Heatmaps reflect log_2_ mean-centred gene expression changes with clustering based on Pearson correlation with average linkage (red = higher expression, black = no change, green = lower expression). Radiosensitive MCF-7 parental cells and their RR derivatives are shown in adjacent heatmaps. For each cell line, untreated baseline controls at 0 h are shown along with both the treated (2 Gy radiation) and untreated controls at 2 h and 8 h. The genes shown are the most differentially expressed in sensitive parental MCF-7 cells, with the largest gene expression differences seen between the untreated controls and the 2 Gy treated cells at 8 h.

Global gene expression analysis was carried out to identify differences between the parental radiosensitive MCF-7 cells and their RR derivatives at 2 and 8 h post-radiation, time points that have previously been used to assess differences in DNA damage response pathways between radiosensitive and RR cells [29,53]. Within the MCF-7 radiosensitive cells, a 2 Gy radiation dose led to the upregulation of genes involved in DNA damage repair, apoptosis and cell cycle arrest; whereas genes involved in cell cycle, gene splicing and transcription were downregulated. The radiation response of the MCF-7 RR cells was different from that of the radiosensitive cells, with an overall reduction in gene expression changes being observed (Figure 5). Similar results were observed within the ZR-751 parental and RR cell lines (Supplementary Figure S2).

### 3.4. MCF-7 Candidate Biomarker Selection

**Figure 6:**
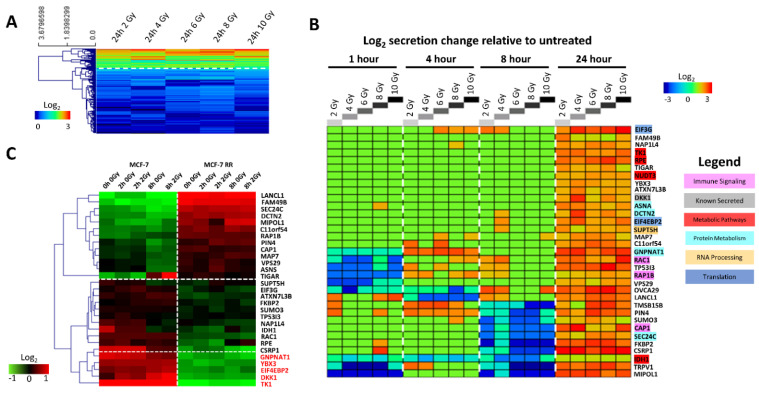
Candidate biomarker selection. (**A**) Protein secretion heatmap showing the log_2_ secretion level of all 159 proteins identified from the radiation-induced secretome across all doses of radiation (2, 4, 6, 8 and 10 Gy) at 24 h. Cluster analysis, performed using Pearson correlation with average linkage, gave rise to two clusters. The upper cluster was found to contain 33 proteins with significantly higher levels of secretion in response to radiation across all doses at 24 h. Heatmap colours indicate log_2_ secretion level as denoted by the colour bar. (**B**) Protein secretion heatmap showing the log_2_ secretion level changes of the 33 proteins from the upper cluster in Figure 3A across all timepoints and radiation doses, normalised to untreated controls at each timepoint. Heatmap colours indicate log_2_ secretion level changes compared to untreated controls at each timepoint as denoted by the colour bar (red = higher expression, green = no change, blue = lower expression). Proteins belonging to pathways found to be enriched in the radiation-induced secretome are highlighted according to the legend. (**C**) Heatmap of log_2_ mean-centred gene expression data from both untreated controls and radiation treated MCF-7 and MCF-7 RR cells, comparing the expression levels of the 33 secreted proteins at the gene level. Clustering was performed using Pearson correlation with average linkage (red = higher expression, black = no change, green = lower expression).

From the 159 proteins which exhibited at least a 50% increase in secretion at 24 h following 2 Gy of radiation, cluster analysis identified 33 proteins that had significantly increased secretion levels (up to 12-fold) at all radiation doses tested (Figure 6A). While a small number of these proteins exhibited increased or decreased secretion levels compared to untreated controls at earlier time points, the secretion levels of the majority of the proteins did not change (Figure 6B). From these 33 proteins, we identified those which were known to be secreted and those which belonged to the previously identified enriched pathways; we hypothesised that it might be these biomarkers that play a role in RT response. Gene expression analysis assessing the levels of these 33 proteins in both MCF-7 and MCF-7 RR cells showed that 5 of the 33 proteins had higher levels of expression in the radiosensitive compared to the RR cells (Figure 6C); similar results were observed within the ZR-751 parental and RR cell lines (Supplementary Figure S3). We chose to focus on these 5 proteins (DKK1, EIF4EBP2, GNPNAT1, TK1 and YBX3) as our candidate biomarkers.

### 3.5. Candidate Biomarker Expression and Intrinsic Sensitivity to Radiation

**Figure 7:**
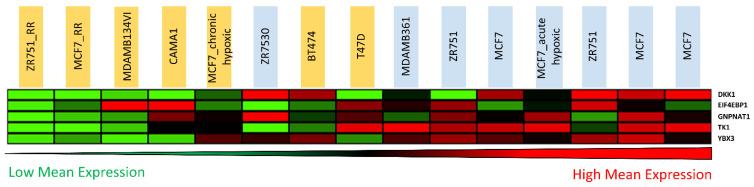
Candidate biomarker expression and intrinsic sensitivity to radiation. Mean-centred gene expression heatmap (red = higher expression, black = no change, green = lower expression) showing the levels of genes encoding the 5 lead candidate biomarkers, ranked left to right by highest mean expression, across a panel of ER^+^ BC cell lines from a public dataset (GSE50811). SF2 values of parental and derived RR cells determined within our lab [29] were combined with SF2 values of a panel of ER^+^ BC cell lines ascertained by others in the literature [19,54,55,56,57]. Cell lines with SF2 values <0.4 and >0.4 were classed as radiosensitive and RR, respectively [58]. The intrinsic radiosensitivity of individual cell lines is indicated by highlighted colour (blue = sensitive, yellow = resistant).

As these five candidate biomarkers had higher inherent gene expression levels within the radiosensitive cells compared to their acquired RR derivatives, we further investigated whether these biomarkers might be linked to intrinsic radiosensitivity. SF2 values (a commonly used experimental indicator of cellular radiosensitivity) of parental and derived RR cells determined within our lab [29] were combined with SF2 values of a panel of ER^+^ BC cell lines ascertained by others in the literature [19,54,55,56,57]. Cell lines with SF2 values <0.4 and >0.4 were classed as radiosensitive and RR, respectively (this threshold has been previously used to define radiosensitivity and radioresistance [58]). Gene expression levels of our five biomarkers were observed to be higher in the more radiosensitive cell lines than in RR models (Figure 7). These results suggest that our candidate biomarkers may be associated with intrinsic radiosensitivity. 

### 3.6. In Vitro and In Vivo Validation of Candidate Biomarkers

To validate the secretomic results and further investigate the potential use of these proteins as biomarkers of radiosensitivity, the secreted and intracellular protein levels of our candidate biomarkers were assessed through WB and IHC, respectively, using both parental radiosensitive and derived RR cell lines. While we initially set out to validate all five candidate biomarkers, we were unable to find suitable antibodies for two of the proteins (EIF4EBP2 and YBX3); we therefore focused on validating DKK1, GNPNAT1 and TK1. 

**Figure 8:**
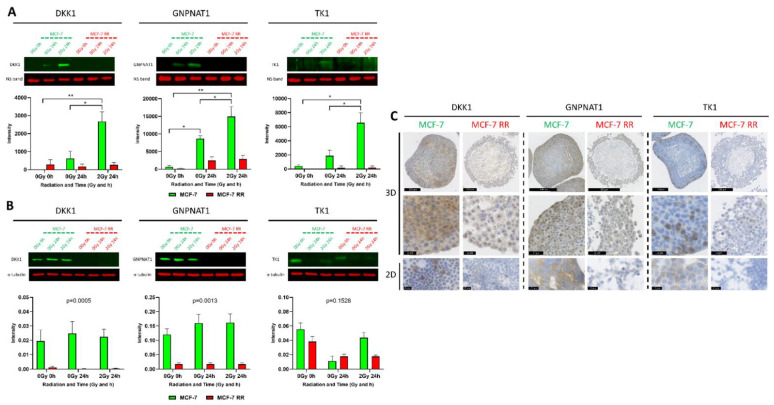
In vitro validation of lead candidate biomarkers. (**A**) WB analysis assessing the secretion levels of lead candidate biomarkers in MCF-7 and MCF-7 RR cell lines using CM samples obtained up to 24 h following 2 Gy of radiation. NS is a non-specific band used to confirm equal loading (One-way ANOVA with Holm–Šídák multiple comparisons test; data expressed as mean ± SEM, *n* = 3, * *p* ≤ 0.05, ** *p* ≤ 0.01). (**B**) WB analysis assessing the intracellular levels of lead candidate biomarkers in whole-cell lysates of MCF-7 and MCF-7 RR cell lines obtained up to 24 h following 2 Gy of radiation (Two-way ANOVA; data expressed as mean ± SEM, *n* = 3). (**C**) IHC assessing the intracellular levels of the lead candidate biomarkers in MCF-7 and MCF-7 RR cells cultured in 2D and 3D environments.

WB analysis was performed using CM samples to assess secreted protein levels from MCF-7 parental and RR cell lines 24 h after the cells had received a single radiation dose of 2 Gy (Figure 8A). Compared to untreated controls, the secretion levels of DKK1, GNPNAT1 and TK1 were significantly increased in MCF-7 cells 24 h after irradiation. In comparison, biomarker levels in the CM samples from untreated and radiation-treated MCF-7 RR cells remained low. Increased levels of secretion of our candidate biomarkers after irradiation was also observed in radiosensitive ZR-751 cells, with no increase in secretion detected in ZR-751 RR cells (Supplementary Figure S4).

Intracellular expression levels of the candidate biomarkers were assessed in both 2D and 3D culture conditions. WB analysis of whole cell lysates of cells cultured in 2D showed that the protein expression levels of DKK1 and GNPNAT1 were significantly higher in the radiosensitive parental MCF-7 cells compared to the RR cells (Figure 8B). Both the 2D ICC and 3D IHC indicated that the parental MCF-7 cells had higher basal levels of the three candidate biomarkers compared to the RR cells (Figure 8C). Similar results were also observed with the ZR-751 radiosensitive and RR cell lines (Supplementary Figure S4).

**Figure 9:**
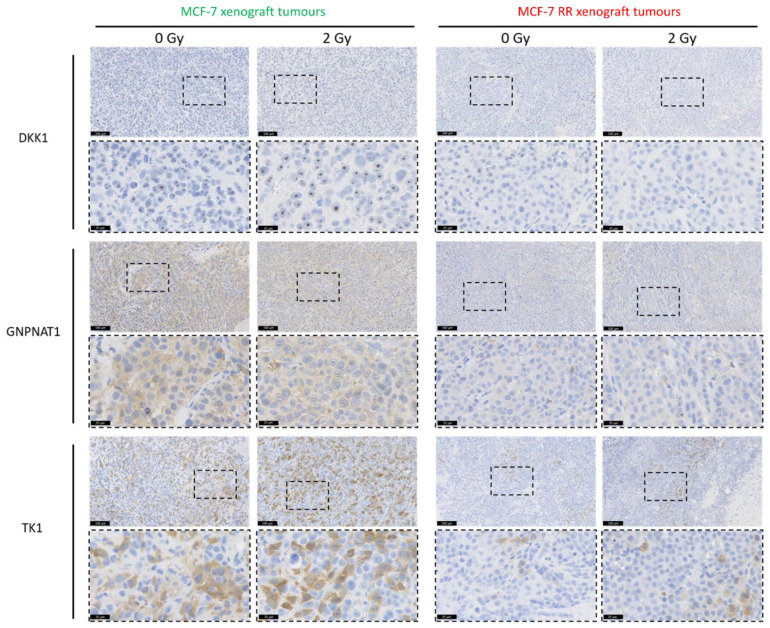
In vivo intracellular levels of lead candidate biomarkers. IHC assessing the intracellular levels of the lead candidate biomarkers in mouse xenograft tumours harvested 24 h after radiation. Representative images taken from five MCF-7 and five MCF-7 RR xenograft tumours.

We further assessed the link between the intracellular levels of these biomarkers and radiosensitivity using mouse xenograft tumours consisting of either MCF-7 parental or MCF-7 RR cells. IHC was performed on these mouse xenograft tumours, which were harvested 24 h post-radiation. Results showed that, while there was no increase in intracellular protein expression levels 24 h after radiation, the intracellular basal levels of the biomarkers were higher in the parental tumours compared to the RR tumours (Figure 9).

### 3.7. Validation in a Retrospective Patient Cohort

**Figure 10:**
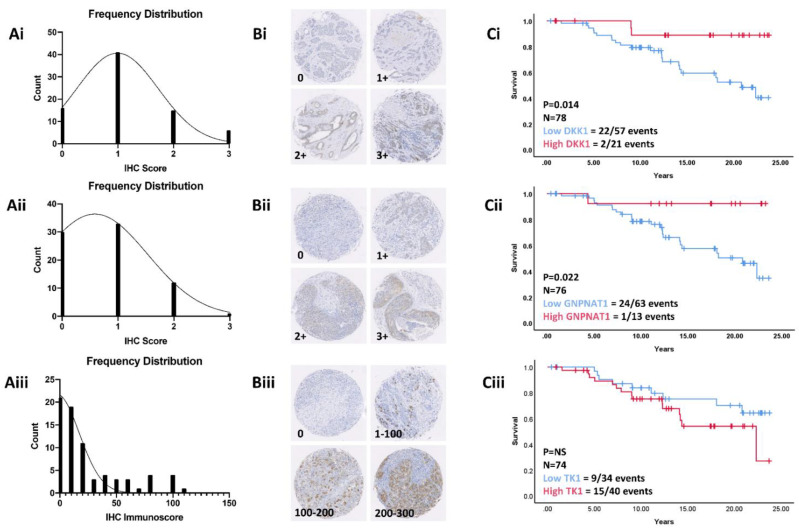
Validation in a retrospective patient cohort. (**A**) Frequency distribution histograms with gaussian regression curves fitted showing distribution of IHC grading histoscores of DKK1 (Ai) and GNPNAT1 (Aii), along with the distribution of IHC grading immunoscores for TK1 (Aiii), across a cohort (*n* = 78) of post-menopausal ER^+^ BC patients treated with surgery and adjuvant RT alone. (**B**) Representative images of IHC staining for DKK1 (Bi), GNPNAT1 (Bii) and TK1 (Biii). (**C**) Kaplan–Meier analysis of recurrence-free survival in relation to DDK1 (Ci), GNPNAT1 (Cii) and TK1 (Ciii) biomarker expression in the patient cohort. Median follow-up is 12.3 years. *p*-value derived from log-rank (Mantel-cox) test.

Previous gene and protein expression analysis indicated that intracellular levels of the candidate biomarkers may be linked with radiosensitivity. Further investigation into whether these candidate biomarkers could predict response to RT was carried out. To do this, we performed IHC to assess the intracellular levels of the three candidate biomarkers using pre-treatment biopsy tissues from ER^+^ BC patients identified in the Breast-Conserving Series. We hypothesised that patients exhibiting higher levels of our candidate biomarkers would have a better response to RT compared to those with lower levels. High intracellular levels of both DKK1 (Figure 10Ci) and GNPNAT1 (Figure 10Cii) were associated with significantly increased recurrence-free survival (DKK1, *p* = 0.014; GNPNAT1, *p* = 0.022), indicating that these two candidate biomarkers have the potential to predict sensitivity to RT. No significant differences in recurrence-free survival were observed in those patients with either low or high intracellular TK1 levels (Figure 10Ciii). High magnification images of the TMA samples are presented in Supplementary Figures S5–S7.

## 4. Discussion

RT is a frequently used curative and palliative treatment for BC. However, for some patients intrinsic and acquired radioresistance can substantially limit the efficacy of RT, ultimately leading to local recurrence, disease progression and/or metastasis. While some studies have investigated tissue-based gene signatures as a way of predicting tumour radiation response [19,20,21], others appreciate the advantages of using blood-based biomarkers as they can be detected less invasively pre-, post- and during treatment; this can allow a patient to be continually monitored. Various clinical studies have explored the utilisation of blood-based biomarkers, such as carbohydrate antigen 15-3 and carcinoembryonic antigen for primary cancer diagnosis and metastatic disease detection [59,60,61,62,63,64], while the association between serum human epidermal growth factor receptor 2 (HER2) levels and tumour HER2 status has also been studied [65,66,67,68]. Pre-clinical studies typically focus on the cancer secretome for the identification of secreted biomarkers. Several secretomic studies have used it to identify biomarkers of aggressive phenotypes or predictors of chemotherapeutic response [23,25,26]. Previous work has also identified secreted biomarkers related to radiosensitivity. One study examined the secretome of BC cells 6 days after treatment with a single dose of 10 Gy, showing that the secretion of cyclophilin A was related to intrinsic radiosensitivity [27]. While this study demonstrated that protein secretion can increase following radiation, and that secreted proteins can relate to radiosensitivity, acute cancer secretome changes after radiation treatment were not assessed. It is these early changes that could potentially be more useful in a clinical setting. As a result of increased clinical interest in the use of blood-based biomarkers to evaluate pre- and on-treatment RT response [11], along with the potential of tissue-based biomarkers to predict tumour radiosensitivity, our study aimed to develop a novel method to identify both secreted and intracellular biomarkers of RT response.

The ER^+^ MCF-7 cell line was chosen as the initial model for biomarker discovery, as it is a well-characterised cell line that has been used in many previous secretomic studies [25,26,69,70,71,72,73]. The first stage of our study involved the acquisition of CM samples from MCF-7 cells for LC-MS. For this, we used the CM of cells cultured in SFM, as serum bovine proteins can dilute the cancer secretome and hinder the identification of secreted proteins due to the close sequence homology of cattle proteins to many human proteins [74]. Even though the effect of serum starvation on cancer cells is disputed [75,76,77,78], studies have demonstrated that culturing cells in SFM does not significantly alter the composition of secreted proteins [79,80] and that cell death is minimised under appropriate culture conditions [25,69,81]. Researchers have recommended that optimal incubation times and cell numbers are needed to diminish the cytosolic protein contamination that arises from cell death. Incubating cells with SFM for up to 30 h, with less than 70% cell confluency, are considered optimal conditions for the acquisition of secretome samples; these culture conditions were followed in all of our experiments. A washing step was also carried out in our study before incubating the cells in SFM; previous studies have demonstrated that washing reduces the contamination of CM with serum proteins and also increases the quantity of secreted proteins isolated, without having any effect on cell growth or viability [82]. 

All CM samples underwent centrifugation to reduce contamination by dead cells and debris, with concentration performed to enrich secreted proteins. This approach has been successfully used previously [83,84] and is necessary because secreted proteins are generally present in low abundance [85]. Control secretome samples were also acquired at each time point to account for the potential effects of serum starvation. To confirm that radiation was not having an effect on cell number or causing significant cell death at 24 h post-treatment, we performed cell counts and LDH assays. LDH is an intracellular enzyme involved in metabolism, if present in the CM it indicates that plasma membrane rupture and cell death has occurred [86]. Our results showed no significant differences in viable cell numbers or LDH levels between the controls and radiation-treated samples. This suggests that radiation-induced changes in secreted protein levels would be a result of changes in secretion processes rather than altered proliferation rates or radiation-induced cell lysis. Our results are in accordance with other secretomic studies that have demonstrated the absence of any significant levels of cell death up to 24 h after treatment with 10 Gy [67,68,69]. 

Our secretome sample preparation method likely led to the co-collection of directly secreted proteins and those secreted through exosome/microvesicle pathways. Using databases such as ExoCarta and Vesipedia we identified that a proportion of our identified secreted proteins had been previously identified within exosomes/microvesicles. Interestingly, exosomal structural proteins were not present within our samples. One possible explanation for this is that exosomes and microvesicles can differ in the composition of their structural proteins including ALIX, TSG101, CD81, CD63 and CD9 [87]. It may be that the primary method of secretion for the proteins we identified using ExoCarta and Vesipedia (which do not differentiate between exosomes and microvesicles) is via microvesicles or even direct secretion rather than in exosomes. Indeed, our current work is focused on answering this important question by repeating our proteomic analysis of secreted samples after applying specific methods to isolate exosomes, microvesicles and directly secreted proteins.

Our secretomic analysis initially focused on CM samples obtained 24 h after irradiation. Cancer patients are typically treated with daily radiation fractions; therefore, the measurement of biomarkers at 24 h after the first dose of fractionated RT might be appropriate in clinical practice. In theory, biomarker levels could be analysed just before daily treatment, that is, 24 h after a patient’s preceding dose. Initial analysis characterised the MCF-7 untreated basal secretome. The number of proteins isolated and the key enriched pathways in which they function (metabolism, carbohydrate metabolism, immune and cytokine signalling and cell cycle regulation) were in agreement with previous studies using various tumour types, including BC cell lines [24,88]. The majority of the proteins detected in the secretome 24 h after radiation differed from those of the basal secretome, specifically those involved in translation, spliceosome and RNA processing, protein metabolism and the proteasome. Proteins involved in some of these pathways have previously been shown to be secreted from BC cells 6 days after a 10 Gy radiation dose [27]. 

In order to identify the most suitable candidate biomarkers to be taken forward for validation, we wanted to identify biomarkers that exhibited a straightforward secretion profile, whereby levels were minimal at earlier time points, then demonstrated a large increase at 24 h, as this might potentially increase the probability of successful validation. Of the proteins that had been identified in the radiation-induced secretome, 33 proteins were found to have significantly increased secretion levels (up to 12-fold) at all radiation doses tested at 24 h, with low secretion at earlier time points.

Further analysis of these 33 proteins focused on their gene expression levels within the MCF-7 radiosensitive and RR cell lines. Initial comparative analysis of the two cell lines showed differences in their gene expression patterns in response to 2 Gy treatment, with radiosensitive cells exhibiting up-regulation of genes involved in DNA damage repair pathways and arrest of the cell cycle, and down-regulation of genes involved in the cell cycle. Similar gene expression changes have been found in other studies using the MCF-7 cell line [89] and patient samples [90]. These recognised radiation-induced gene expression changes did not occur in the RR cells. DNA damage repair pathways play a crucial role in the response of cells to radiation; previous studies have also shown there to be differences in the expression of DNA damage related genes between radiosensitive and RR cell lines [53]. Given the differences in response to radiation, we proposed that any of our 33 secretomic candidate biomarkers that were differentially expressed between the sensitive and resistant cell lines could hold value as biomarkers of RT response or acquired radioresistance. Gene expression analysis assessing the 33 proteins showed that DKK1, EIF4EBP2, GNPNAT1, TK1 and YBX3 had higher expression levels in the radiosensitive cells. Further evidence of a relationship between the gene expression levels of these 5 candidate biomarkers and radiosensitivity was shown in a panel of ER^+^ cells, with the more radiosensitive cells expressing higher levels of the candidate biomarkers. Validation experiments focusing on DKK1, GNPNAT1 and TK1 showed that these biomarkers were secreted in response to radiation treatment, but only in radiosensitive cells. These results were recapitulated in a second ER^+^ cell line (ZR-751). Results from the in vitro and in vivo experiments indicated that intracellular protein levels of these three biomarkers may also be associated with radiosensitivity. Further evidence of the biomarkers potential to predict RT response was seen through assessing intracellular protein expression levels using samples from the Breast-Conserving Series. Here, survival analysis identified that patients with higher intracellular DKK1 and GNPNAT1 expression levels were associated with significantly increased recurrence-free survival.

Prior studies have linked our three lead candidate biomarkers with cancer. DKK1 is a soluble antagonist of Wnt/β-catenin signalling [91]. Previous work has suggested that Wnt signalling and DKK1 are involved in bone metastasis [92] and that DKK1 can stimulate osteoclast activity and inhibit the production and differentiation of osteoblasts. Inhibition of the effects of Wnt on the bone can help generate a microenvironment that allows tumours to expand [93]. DKK1′s role in stimulating osteolytic metastases has been established in investigations of multiple myloma-associated bone disease [94,95], with differing studies also supporting the role of DKK1 in BC bone metastasis. Serum concentrations of DKK1 have also been shown to be increased in BC patients; moreover, patients with bone metastases were shown to have significantly increased serum DKK1 levels when compared to non-metastatic BC patients [96]. Elevated serum DKK1 concentrations have also been correlated with more advanced disease stage and grade of BC, along with shorter recurrence-free and overall survival times [97]. A further study demonstrated that although DKK1 was present in 70% of BC tissues, it could be identified in all patients using serum samples [96]. Altogether, these studies show that DKK1 is a promising intracellular and secreted biomarker for assessing BC prognosis. 

GNPNAT1 is an enzyme involved in the hexosamine biosynthetic pathway (HBP). The HBP produces UDP-N-acetylglucosamine (UDP-GlcNAc), which is thought to be an essential nutrient sensor [98]. UDP-GlcNAc itself is used as substrate in glycosylation reactions; these post-translational changes are highly altered in tumour cells and can regulate the function of proteins involved in various tumour-associated processes such as gene regulation, metabolism, cell signalling and epithelial-to-mesenchymal-transition [98]. GNPNAT1 expression has been linked with prognosis in prostate cancer; higher expression levels have been associated with a lower risk of biochemical recurrence [99], whereas lower levels are typically seen in advanced, castrate-resistant prostate cancer when compared to localised disease [100]. Studies have demonstrated that GNPNAT1 is upregulated in lung adenocarcinoma tissues compared to normal tissues [101,102], with Liu et al. concluding that this protein may have potential as a prognostic biomarker [101]. Our results indicate that GNPNAT1 may additionally have a role to play in BC. This is in line with other recent studies which have demonstrated that elevated GNPNAT1 gene expression levels are present in BC tissue samples [103]. 

TK1 is involved in cell cycle regulation through the production of thymidine monophosphate, an essential requirement for DNA replication [104,105]. TK1 has been identified in extracellular vesicles from numerous cancer types [106,107,108,109]. Some studies have suggested that it could be used as a proliferation biomarker [110] with both diagnostic and prognostic potential [104,111]. In BC, increased intracellular TK1 expression has been correlated with disease grade and stage [112], with serum levels having been investigated for monitoring treatment responses [113] and for predicting the risk of developing distant and/or regional recurrence post-surgery [114]. 

In BC, RT is traditionally carried out in the adjuvant setting, after breast-conserving surgery and sometimes after mastectomy to eliminate any residual cancer cells left behind after surgery. While our results are promising, there are potential limitations to their translatability to the clinic. A potential issue is that there could be differences in secreted biomarker levels when RT is given neoadjuvantly to shrink in situ cancers compared with levels seen after post-operative adjuvant RT dealing with residual tumour cells. However, RT does also have a role in the management of BC in the neoadjuvant setting, where it can be combined with chemotherapy in patients with locally advanced cancer [115,116,117,118,119,120,121]. Neoadjuvant RT alone has been used for the treatment of BCs that are unsuitable for primary conservative surgery [122]. There is also increasing interest in the use of neoadjuvant accelerated partial breast irradiation alone to help reduce treatment-related morbidities associated with external beam irradiation [123,124]. Recent work has additionally shown that neoadjuvant RT alone may significantly increase disease-free survival without decreasing overall survival in patients with early-stage BC; these results were most evident for ER^+^ BC patients [125]. As our study used ER^+^ BC cell lines, our results may be of particular utility to early-stage patients suffering from this BC subtype. Recent work has also shown that neoadjuvant RT alone, followed by radical surgery, is a feasible treatment option and is associated with good long-term locoregional control [126]. Therefore, while pre-surgical RT is not currently the standard treatment option for patients, neoadjuvant RT has the potential to challenge the current treatment paradigm. This BC treatment strategy will ultimately require biomarkers, such as ours, that can predict and monitor RT response. 

Although previous studies have shown that each of our candidate biomarkers is secreted from BC cells [106], with some of them linked to BC prognosis, ours is the first study to describe a link between the intracellular/secreted levels of these biomarkers and radiosensitivity. Whilst our initial model for secreted biomarker discovery was only performed using the MCF-7 cell line, our secretomic results have been comprehensively validated using two different ER^+^ cell lines. Although these results are promising, additional work is now needed to assess whether these biomarkers can be detected in blood samples using animal models. Following on from our successful use of BC xenograft tumours and patient tissues from the Breast-Conserving Series, we will now look to investigate the biomarker’s ability to predict radiosensitivity in larger patient cohorts. Furthermore, experiments will be needed to investigate the mechanisms of biomarker secretion and elucidate what roles these biomarkers play in cellular radiosensitivity. Although our study is particularly focused on BC, it is possible that the biomarkers we have identified are not BC-specific but may be more generic measures of tumour radiosensitivity. The methods we have used to identify biomarkers of radiation response are equally applicable to other solid tumours; future studies could therefore utilise our validated methods for biomarker discovery in other cancer types.

## 5. Conclusions

For clinicians to be able to deliver biologically adapted, personalised RT for BC patients they must be able to stratify patients based on individual tumour radiosensitivity before commencing treatment. Clinicians should also be able to monitor RT responses during treatment. To begin to address these clinical needs we developed an integrated secretomic and transcriptomic approach using both radiosensitive and RR cell lines to identify biomarkers of radiation sensitivity and response. To our knowledge, we are the first to report the use of secretomic experiments to identify radiation-induced BC secreted biomarkers that are released within 24 h of treatment. Furthermore, we showed that differential biomarker secretion, gene expression and intracellular protein levels can indicate cellular radiosensitivity. Initial validation using clinical samples also suggested that two of our selected candidate biomarkers have the potential to predict RT outcomes in ER^+^ BC patients. For any of these intracellular/secreted candidate biomarkers to be used in the clinic, further research will have to prove their validity and demonstrate their ability to improve outcomes or refine patient selection for RT. The incorporation of individual biomarkers and/or signatures with advanced radiation delivery techniques, already available in the clinic, would enable the development of a precision medicine platform that could significantly improve the efficacy of RT in the treatment of BC patients. 

## Data Availability

The gene expression datasets generated and/or analysed during the current study are available in the NCBI’s Gene Expression Omnibus [44]; accessible with GEO Series accession number GSE120798. All secretomic data are available on the PRoteomics IDEntifications Database [35,36]; accessible with the project accession number PXD027572.

## Supplementary Materials

The following are available online at https://www.mdpi.com/article/10.3390/jpm11080796/s1. Supplementary Figure S1. Cell numbers and LDH cytotoxicity assays at 24 h post-radiation treatment. (**A**) Cell counts using trypan blue exclusion were performed with MCF-7 and ZR-751 parental and RR cell lines to confirm that no changes in proliferation or cell death were occurring after treatment with a single dose of up to 10 Gy radiation (one-way ANOVA with the Holm–Šídák multiple comparisons test, comparing only values within each cell line; data expressed as mean ± SEM, *n* = 3). (**B**) LDH cytotoxicity assays were performed with MCF-7 and ZR-751 parental and RR cell lines to confirm that no cell death was occurring after treatment with 2 Gy of radiation (unpaired *t*-test performed on the control and treated cells for each cell line; data expressed as mean ± SEM, *n* = 3). Supplementary Figure S2. ZR-Z51 and ZR-751 RR gene expression changes associated with response to radiation. Heatmaps reflect log_2_ mean-centred gene expression changes with clustering based on Pearson correlation with average linkage (red = higher expression, black = no change, green = lower expression). Radiosensitive ZR-751 parental cells and their RR derivatives are shown in adjacent heatmaps. For each cell line, untreated baseline controls at 0 h are shown along with both treated (2 Gy radiation) and untreated controls at 2 h and 8 h. The 2 h 2 Gy ZR-751 sample failed in sequencing and was removed from further analysis. The genes shown are the most differentially expressed in sensitive parental MCF-7 cells, with the largest gene expression differences seen between the untreated controls and the 2 Gy treated cells at 8 h. Supplementary Figure S3. Gene expression levels of the 33 candidate biomarkers within ZR-751 parental and RR cell lines. Heatmap of log_2_ mean-centred gene expression data from both untreated controls and radiation-treated parental ZR-751 and ZR-751 RR cells. Clustering was performed using Pearson correlation with average linkage (red = higher expression, black = no change, green = lower expression). Supplementary Figure S4. In-lab validation of lead candidate biomarkers in the ZR-751 cell line. (**A**) WB analysis assessing the secretion levels of lead candidate biomarkers in ZR-751 and ZR-751 RR cell lines using CM samples obtained up to 24 h following 2 Gy of radiation. NS is a non-specific band used to confirm equal loading (One-way ANOVA with Holm–Šídák multiple comparisons test; data expressed as mean ± SEM, *n* = 3, * *p* ≤ 0.05, *** *p* ≤ 0.001, **** *p* ≤ 0.0001). (**B**) WB analysis assessing the intracellular levels of lead candidate biomarkers in whole-cell lysates of ZR-751 and ZR-751 RR cell lines obtained up to 24 h following 2 Gy of radiation (Two-way ANOVA; data expressed as mean ± SEM, *n* = 3). (**C**) IHC assessing the intracellular levels of the lead candidate biomarkers in ZR-751 and ZR-751 RR cells cultured in 2D and 3D environments. Supplementary Figure S5: High magnification TMA images stained for DKK1. Images are taken from those tissues presented in Figure 10. Supplementary Figure S6: High magnification TMA images stained for GNPNAT1. Images are taken from those tissues presented in Figure 10. Supplementary Figure S7: High magnification TMA images stained for TK1. Images are taken from those tissues presented in Figure 10. Supplementary Table S1. RNA quality of the samples used for gene expression analysis. RNA integrity numbers (RIN) for the gene expression analysis samples. Supplementary Table S2. Clinicopathological data from 80 patients within the Breast-Conserving Series were used to investigate whether the candidate biomarkers could predict response to RT. Supplementary Table S3. List of proteins identified in each pathway from the untreated MCF-7 cell secretome. In total, 318 proteins were identified in the untreated basal MCF-7 secretome. Proteins involved in the significantly enriched pathways identified from the KEGG and Reactome databases are shown. Supplementary Table S4. List of proteins identified that exhibited at least a 50% increase in secretion level following 2 Gy of radiation compared with 24 h untreated controls. A proportion of the secreted proteins were involved in immune signalling, metabolism, translation, RNA processing and the proteasome.
