# Supplementary material for: A Novel Approach for the Discovery of Biomarkers of Radiotherapy Response in Breast Cancer
Source: J Pers Med. 2021 Aug 14;11(8):796. doi: 10.3390/jpm11080796 (PMC8399231; doi:10.3390/jpm11080796)
Supplement: Supplementary file 1 [file jpm-11-00796-s001.zip › Supplementary Table S1.pdf]

Sample	RIN	Sample	RIN
MCF-7 0h 0Gy	9.9	ZR-751 0h 0Gy	9.8
MCF-7 2h 0Gy	9.9	ZR-751 2h 0Gy	9.8
MCF-7 2h 2Gy	9.9	ZR-751 2h 2Gy	9.7
MCF-7 8h 0Gy	9.8	ZR-751 8h 0Gy	9.7
MCF-7 8h 2Gy	9.8	ZR-751 8h 2Gy	9.7
MCF-7 RR 0h 0Gy	10	ZR-751 RR 0h 0Gy	10
MCF-7 RR 2h 0Gy	10	ZR-751 RR 2h 0Gy	10
MCF-7 RR 2h 2Gy	10	ZR-751 RR 2h 2Gy	10
MCF-7 RR 8h 0Gy	10	ZR-751 RR 8h 0Gy	10
MCF-7 RR 8h 2Gy	10	ZR-751 RR 8h 2Gy	10

Supplementary Table S1. RNA quality of the samples used for gene expression analysis. RNA integrity numbers (RIN) for the gene expression analysis samples.
