# Supplementary material for: A Novel Approach for the Discovery of Biomarkers of Radiotherapy Response in Breast Cancer
Source: J Pers Med. 2021 Aug 14;11(8):796. doi: 10.3390/jpm11080796 (PMC8399231; doi:10.3390/jpm11080796)
Supplement: Supplementary file 1 [file jpm-11-00796-s001.zip › Supplementary Table S2.pdf]

**Patient Age**	Mean 65.2 (range 35-91)
**Grading Characteristic**	**Number of Patients**
**Histological Grade**	
1	30
2	37
3	13
**ER Status (Allred Score)**	
6	18
7	42
8	20
**PgR Status**	
6	10
7	21
8	20
Unknown	29
**Lymph Node Status**	
Positive	18
Negative	62
**T stage (TNM)**	
T1	51
T2	29
**Surgery**	
Breast conserving surgery (WLE)	80
Mastectomy	0
**Adjuvant Treatment**	
Chemotherapy	0
Endocrine therapy	0
Radiotherapy	80

Supplementary Table S2. Clinicopathological data from 80 patients within the Breast-Conserving Series that were used to investigate whether the candidate biomarkers could predict response to RT.
