# Supplementary material for: A Novel Approach for the Discovery of Biomarkers of Radiotherapy Response in Breast Cancer
Source: J Pers Med. 2021 Aug 14;11(8):796. doi: 10.3390/jpm11080796 (PMC8399231; doi:10.3390/jpm11080796)
Supplement: Supplementary file 1 [file jpm-11-00796-s001.zip › Supplementary Table S3.pdf]

All Secreted Proteins	Secreted Proteins in the known Interactome	Metabolic Pathways	Carbohydrate Metabolism	Cell Cycle	Immune
LRRCC1	RPL11	ABAT	ACO2	DYNC1LI2	ACLY
ASS1	RPSA	ACACA	ADH5	GORASP2	ACTB
CTSB	GART	ACLY	ALDOA	LMNB1	ACTG1
CKAP4	TKT	ACO2	ALDOC	MAPRE1	ALDOA
PLS3	GPI	ADH5	CS	NUP37	ALDOC
KIF5A	SUMO1	ADI1	DLD	PCNA	APEH
TBCA	PSMA7	ADK	ENO1	PPP1CB	APRT
CTSD	TPI1	ADSS	ENO2	PPP1CC	B2M
PFN2	LSM2	AKR1A1	ESD	PSMA7	BLMH
MIPOL1	ACACA	ALDH9A1	FBP1	PSMB1	BOLA2
ALDOA	KRT8	ALDOA	FBP2	RBBP4	CALM1
DLD	IDI1	ALDOC	FH	RCC2	CFL1
SORD	RAN	APRT	G6PD	RRM2	CKAP4
TPD52L2	HSPD1	ASS1	GAPDH	SFN	COTL1
MIF	GNB2L1	ATIC	GCSH	SUMO1	CPPED1
MAT2B	FH	BLVRB	GOT2	UBE2I	CSTB
KRT19	FDPS	BPNT1	GPI	UBE2V2	CTSB
ADI1	HSPA5	CBR1	IDH1	YWHAB	CTSD
PA2G4	FASN	CBR3	MDH1	YWHAE	DYNC1LI2
LMAN2	MDH1	CKMT1A	MDH2	YWHAG	EIF4A1
DDB1	CS	CMBL	ME1	YWHAH	FABP5
EML2	SHMT2	CNDP2	PGAM1	YWHAQ	FKBP1A
F11	SHMT1	COX17	PGD	YWHAZ	FLNA
HMGN1	SUMO2	COX4I1	PGK1		FLNB
ALDOC	TXNRD1	CS	PGLS		GDI2
PGM2	ADI1	DCTPP1	PGP		GPI
VIM	PKM	DLD	PKM		GSTO1
YWHAH	PGK1	DUT	PSAT1		GYG1
SH3BGRL	APRT	ENO1	SHMT1		HEBP2
DDAH1	ALDOA	ENO2	SHMT2		HIF1A
GNPNAT1	MAT2B	ENOPH1	TALDO1		HSPA5
SHMT2	ACTB	FAH	TKT		HSPA8
TARS	PGD	FAHD1	TPI1		IDH1
GORASP2	MDH2	FASN			KIF5A
SERF2	ESD	FBP1			KRT1
DNAJC9	TXN	FBP2			LMNB1
TPT1	PGAM1	FDPS			LTA4H
LDHA	GCSH	FH			MIF
RPL11	EIF4A1	G6PD			MSN
SFN	AIMP1	GAPDH			MYH9
MTPN	PNP	GART			NIT2

HMGN2	G6PD	GCLC			NPEPPS
G6PD	UBE2V2	GCSH			NUP37
DDAH2	TALDO1	GNPDA1			PA2G4
RBBP9	GSR	GOT2			PDAP1
CALM1	NAGK	GPI			PDIA3
PKM	GCLC	GSS			PGAM1
ACTG1	ASS1	GYG1			PGM2
NUP37	ENO2	HPRT1			PKM
KRT18	ADSS	IDH1			PNP
ANXA3	EIF2S2	IDI1			POLR2H
LAMB1	IDH1	IMPA1			PPIA
PPP2R4	ACTN4	LAP3			PRDX6
SH3BGRL3	ME1	LDHA			PSMA7
FDPS	HSPA8	LTA4H			PSMB1
ISOC1	ENO1	MAT2A			RAPGEF4
CBR3	GDI2	MAT2B			S100A11
DNASE2	FBP1	MDH1			SOD1
HIST1H4A	LDHA	MDH2			SUMO1
KRT8	FAH	ME1			TALDO1
EIF4A1	ALDH9A1	MVD			TCEB2
HN1L	PCNA	NAGK			THOP1
APEH	GNPDA1	NANS			TXN
HEBP2	GAPDH	NME2			UBE2V2
ME1	CFL1	OAT			VCL
GSS	PPIH	PFAS			VIM
CFL1	PFAS	PGAM1			YWHAB
FANCD2	ACLY	PGD			YWHAZ
KRT2	GDI1	PGK1			
CMBL	UBE2I	PGLS			
PFN1	TPT1	PGM2			
PRRC2C	PGM2	PGP			
PPIH	MAPRE1	PKM			
DTD1	FBP2	PNP			
FASN	NUP37	POLR2H			
PGK1	ACTG1	PRDX6			
NAPA	DUT	PSAT1			
GART	WDR5	RRM2			
FBP1	KRT18	SHMT1			
NUDT2	EIF2A	SHMT2			
C1orf123	YWHAG	SORD			
GDI1	HIF1A	TALDO1			
TXN	CTSB	TKT			

WDR5	ABAT	TPI1			
REXO2	CALU	TSTA3			
TMSB4X	CSTB				
NAGK	AKR1A1				
TRIM28	GLRX				
NIT2	PARK7				
GOT2	KIF5A				
TTN	HPRT1				
SCRN1	B2M				
HRSP12	HMGN1				
CS	ALB				
S100A11	RBBP4				
PPIA	PPIL1				
IDI1	POLR2H				
OVCA2	YWHAZ				
PNP	WDR12				
TP53I3	YWHAB				
PSMA7	KRT2				
FH	CMPK1				
GCSH	EIF4H				
OTUB1	RRM2				
ESD	YWHAQ				
CLIC1	CTTN				
PGAM1	NME2				
CSDE1	GOT2				
PHPT1	TCEB2				
UBE2I	FABP5				
PDLIM1	KRT19				
ENO1	TTN				
EIF2A	KRT10				
AAMP	LSM3				
PROSC	ZNF300				
UBE2L3	PRDX6				
GLO1	PPP2R4				
PPIB	HSPH1				
PCBD1	GRPEL1				
NLN	KRT1				
COX4I1	GGCT				
DPYSL2	LAP3				
MDH1	NIT2				
GNPDA1	CTSD				
GDI2	COX17				

FLNB	HEBP2				
YWHAZ	TARS				
LSM3	TMSB4X				
BLMH	PPP1CB				
KRT10	YWHAE				
GPI	CNDP2				
TKT	CKAP4				
PIR	HSPA4				
PGLS	CPPED1				
RBBP4	LTA4H				
WDR12	PPP1CC				
RAN	PIR				
CMPK1	PA2G4				
ACACA	PEBP1				
RAPGEF4	RCC2				
NME2	NUDT2				
CSTB	PFN2				
C19orf10	SFN				
LTA4H	LMAN2				
PRDX6	DYNC1LI2				
RRM2	VPS29				
SPINT2	RAPGEF4				
FAHD1	TFRC				
DENR	YWHAH				
PGD	FANCD2				
HSPB1	OLA1				
HMCN1	EIF6				
HSPA4	FAHD1				
SUMO2	MSN				
CTTN	PFN1				
HIF1A	SOD1				
TXNL1	ADK				
GRPEL1	GSTO1				
UBE2V2	GSTM3				
LSM2	PSAT1				
TFRC	FLNB				
ACTB	CBR3				
TXNRD1	FLNA				
YWHAG	MTPN				
ADSS	GLO1				
HSPD1	ANXA5				
GCLC	ACO2				

DBI	PPIA				
RPSA	KARS				
GLOD4	PLEC				
PPIF	ERH				
NRBP1	CALM1				
PCMT1	COX4I1				
PTMA	ENSG00000160200				
ANXA5	TMA7				
FKBP4	PLS3				
NUTF2	DTD1				
FBP2	PDIA3				
TPI1	NPEPL1				
PCNA	DNPEP				
NEDD8-MDP1	WDR1				
ALB	RPL19				
EIF4H	ATIC				
EPPK1	PSMB1				
FKBP1A	MVD				
TSTA3	NUTF2				
SUMO1	ENOPH1				
HSPA4L	MAT2A				
HSPB11	PGLS				
LAP3	ADH5				
PPIL1	DLD				
OLA1	GSS				
NT5DC1	GNPNAT1				
ENSG00000124208	ALDOC				
DNPEP	VCL				
GLRX	HSPB1				
PGP	ARHGDIA				
TSN	DDB1				
APRT	FKBP4				
GNB2L1	APEX1				
ECI1	HMGN2				
ENO2	HSPA4L				
DSTN	TRIM28				
SOD1	AAMP				
SELENBP1	PPIL3				
ALDH9A1	MIF				
FAH	LAMB1				
NIF3L1	KRT9				
KARS	VIM				

ERH	DCTPP1				
LXN	LMNB1				
KRT1	PPP1CA				
ZNF300	OAT				
MYH9	NAPA				
ABAT	MYH9				
S100A13	DNASE2				
ENSG00000160200	PPIB				
CNDP2	DNAJC9				
AKR1A1	CBR1				
EIF2S2	CLIC1				
VPS29	SORD				
HN1	PTMA				
LGALS3BP	PEPD				
PPP1CA	SH3BGRL				
THOP1	DTD2				
GSTM3	RBBP9				
FLNA	DENR				
TMA7	DSTN				
PPP1CB	ACYP1				
GSR	TXNL1				
ARHGDIA					
MAPRE1					
GAPDH					
RPL19					
EIF6					
HSPH1					
PDIA3					
TATDN1					
YWHAE					
COTL1					
MAT2A					
PEBP1					
CNPY2					
TCEB2					
BOLA2					
MDH2					
SHMT1					
YWHAQ					
DUT					
FABP5					
NANS					

LMNB1					
HPRT1					
YWHAB					
WDR1					
PPP1CC					
CALU					
ADH5					
NUDT1					
KRT9					
ACYP1					
ATIC					
MAP4					
TXNDC17					
IDH1					
PPIL3					
TFF1					
RCC2					
B2M					
RNPEP					
DTD2					
PTRHD1					
TMSB10					
YTHDF2					
GSTO1					
PLEC					
ACLY					
MVD					
ENOPH1					
PSAT1					
PSMB1					
VAPB					
TALDO1					
IMPA1					
DYNC1LI2					
UFC1					
NPEPPS					
CBR1					
VCL					
PEPD					
PROCR					
POLR2H					
CLIC4					

OAT					
DPP3					
PDAP1					
PFAS					
GGCT					
BPNT1					
CPPED1					
CKMT1A					
PARK7					
BASP1					
COX17					
NR2C2AP					
APEX1					
DCTPP1					
GYG1					
ACO2					
ADK					
BLVRB					
AIMP1					
HSPA8					
NPEPL1					
MSN					
HSPA5					
ACTN4					
GMFB					

Supplementary Table S3. **List of proteins identified in each pathway from the untreated MCF-7 cell secretome.** 318 proteins were identified in the untreated basal MCF-7 secretome. Proteins involved in the significantly enriched pathways identified from the KEGG and Reactome databases are shown.
