# Supplementary material for: A Novel Approach for the Discovery of Biomarkers of Radiotherapy Response in Breast Cancer
Source: J Pers Med. 2021 Aug 14;11(8):796. doi: 10.3390/jpm11080796 (PMC8399231; doi:10.3390/jpm11080796)
Supplement: Supplementary file 1 [file jpm-11-00796-s001.zip › Supplementary Table S4.pdf]

All Secreted Proteins	Secreted Proteins in the known Interactome	Metabolic Pathways	Immune	Protein Metabolism & Proteasome	RNA Processing	Translation
ASNS	RPS8	ADH5	ACTR1A	ACTR1A	DNAJC8	EIF3G
ASS1	RPL30	ADK	ANXA2	ARF1	HNRNPC	GARS
RPSA	RPL10A	AHCY	ARF1	ASNS	LSM6	KARS
PITHD1	RPSA	AKR1A1	ARPC1B	DCTN2	LSM8	MRPL12
BOLA1	LSM6	ARF1	CAP1	EIF3G	NUDT21	RPL10A
PCMT1	HNRNPC	ASNS	CFL1	GARS	PSME2	RPL19
AHNAK	S100A10	ASS1	CLTA	GNPNAT1	PTBP1	RPL30
MIPOL1	COL3A1	BLVRB	COL1A1	HNRNPC	RAN	RPS8
ARPC1B	PGK1	CAD	COL3A1	IGFBP5	RPL10A	RPSA
FUBP1	ASS1	COX7C	COTL1	KARS	RPL19	SRP14
SORD	ESD	DAK	DAK	MRPL12	RPL30	
TPD52L2	RAN	DCXR	DCD	NUDT14	RPS8	
EIF3G	TALDO1	DNPH1	DCTN2	PCMT1	RPSA	
AHCY	EIF3G	ESD	FSCN1	PFDN1	SUPT5H	
CLTA	PTBP1	GAPDH	GSTP1	PSME2	THUMPD1	
HMGN4	SPARC	GCLM	IDH1	RAB8A	XPO1	
XPO1	RPE	GSTM3	NIT2	RPL10A		
UBE2S	ZYX	GSTP1	PDXK	RPL19		
RAB8A	SEC22B	IDH1	PGM1	RPL30		
LSM8	DCTN2	KARS	PIN1	RPS8		
IGFBP5	IDH1	NAMPT	PSME2	RPSA		
TBCB	COL12A1	NUDT3	RAC1	SEC22B		
SHROOM2	GARS	PDXK	RALA	SEC24C		
SH3BGRL	RAC1	PGD	RAP1B	SRP14		
COL1A1	CAD	PGK1	S100A9	TBCB		
COX7C	TLN1	PGM1	SEC22B	TLN1		
FKBP2	ARF1	PPP1CC	SEC24C	TRIM25		
GNPNAT1	NUDT21	PSME2	SRP14	UBE2S		
SEC24C	XPO1	RAN	TALDO1	UCHL3		
FAM129B	ARPC1B	RPE	TRIM25			
NUDT21	SRP14	RPL10A	TRIM29			
HDDC2	CXCL12	RPL19	UBE2S			
TLN1	DCXR	RPL30				
TK1	DNAJC8	RPS8				
KARS	PSMD13	RPSA				
DNPH1	NIT2	SEC24C				
RPS8	ADK	SORD				
EFNB2	PGM1	TALDO1				
C11orf54	DAK	TK1				
PSME2	CAP1	VAPB				
ARHGAP1	RAB8A					

FSCN1	ARHGAP1					
AKR1A1	UBE2D3					
DNAJC8	ACTR1A					
CSRP1	FAM49B					
KRT7	EFNB2					
VPS29	CFL1					
DCTN2	SUPT5H					
CD3EAP	FKBP2					
RPL10A	EIF6					
NAP1L4	AHNAK					
C20orf27	GSTP1					
GSTM3	GAPDH					
MRPL12	PGD					
CFL1	ENSG00000196689					
WIBG	ADH5					
THUMPD1	CLIC1					
S100A9	COX7C					
LGALS8	FUBP1					
NAA50	YBX3					
YBX3	WDR1					
TRIM29	PPP1CC					
S100A10	EIF2A					
ENSG00000196689	IGFBP5					
PGK1	AKR1A1					
GAPDH	PDXK					
RPL19	RNPEP					
EIF6	SEC24C					
ZYX	ANXA2					
TATDN1	CD3EAP					
NAMPT	CSRP1					
COTL1	WIBG					
NUDT14	PIN4					
PAWR	GCLM					
CLEC3B	C11orf54					
DCD	NUDT3					
FAM49B	PFDN1					
HDHD2	DKK1					
COL3A1	VAPB					
MCAM	PDCD6IP					
PDCD6IP	MRPL12					
PDXK	TK1					
MAP7	ABRACL					
DCXR	COTL1					

SEC22B	KARS					
TCEA2	MCAM					
PSMD13	GSTM3					
NIT2	FSCN1					
GARS	SORD					
UBE2D3	NAA50					
ABRACL	LSM8					
PIN1	UBE2S					
UCHL3	PDLIM7					
WDR1	RPL19					
RPE	COL1A1					
PPP1CC	ASNS					
PIN4	RAP1B					
ADH5	CLTA					
GSTP1	PSME2					
OVCA2	AHCY					
TP53I3	TRIM25					
CXCL12	RALA					
GCLM	MGMT					
C12orf5	TCEA2					
RAC1	PIN1					
IDH1	SUB1					
CAD	C12orf5					
SAFB2	CORO1B					
ESD	PCNP					
CLIC1	UBQLN1					
PTBP1	SH3BGRL					
MGMT	HDHD2					
CAP1	S100A14					
SRP14	NAP1L4					
ARF1	NAMPT					
DAK	PITHD1					
RNPEP	NUDT14					
PDLIM1	PCMT1					
HNRNPC	SUMO3					
LSM6	S100A9					
COL12A1						
ENSG00000243667						
EIF2A						
SUMO3						
PCNP						
VAPB						
TALDO1						

PFDN1						
ENSG00000158427						
ACTR1A						
RPL30						
EIF4EBP2						
RAN						
LANCL1						
SMAP1						
CORO1B						
MT1E						
RAP1B						
XPNPEP1						
S100A14						
PGD						
NUDT3						
SUB1						
PDLIM7						
HMCN1						
ATXN7L3B						
DKK1						
CRABP2						
SPARC						
TRIM25						
ADK						
ANXA2						
BLVRB						
RALA						
SUPT5H						
C6orf211						
UBQLN1						
PGM1						
TIGAR						

Supplementary Table S4. **List of proteins identified in each pathway from the radiation-induced breast cancer cell secretome.** Functional analysis led to the identification of 159 proteins exhibiting at least a 50% increase in secretion level following 2 Gy of radiation compared with 24 h untreated controls. Significantly enriched pathways from the KEGG and Reactome databases were identified.
