# Supplementary figures and images for: A Novel Approach for the Discovery of Biomarkers of Radiotherapy Response in Breast Cancer
Source: J Pers Med. 2021 Aug 14;11(8):796. doi: 10.3390/jpm11080796 (PMC8399231; doi:10.3390/jpm11080796)

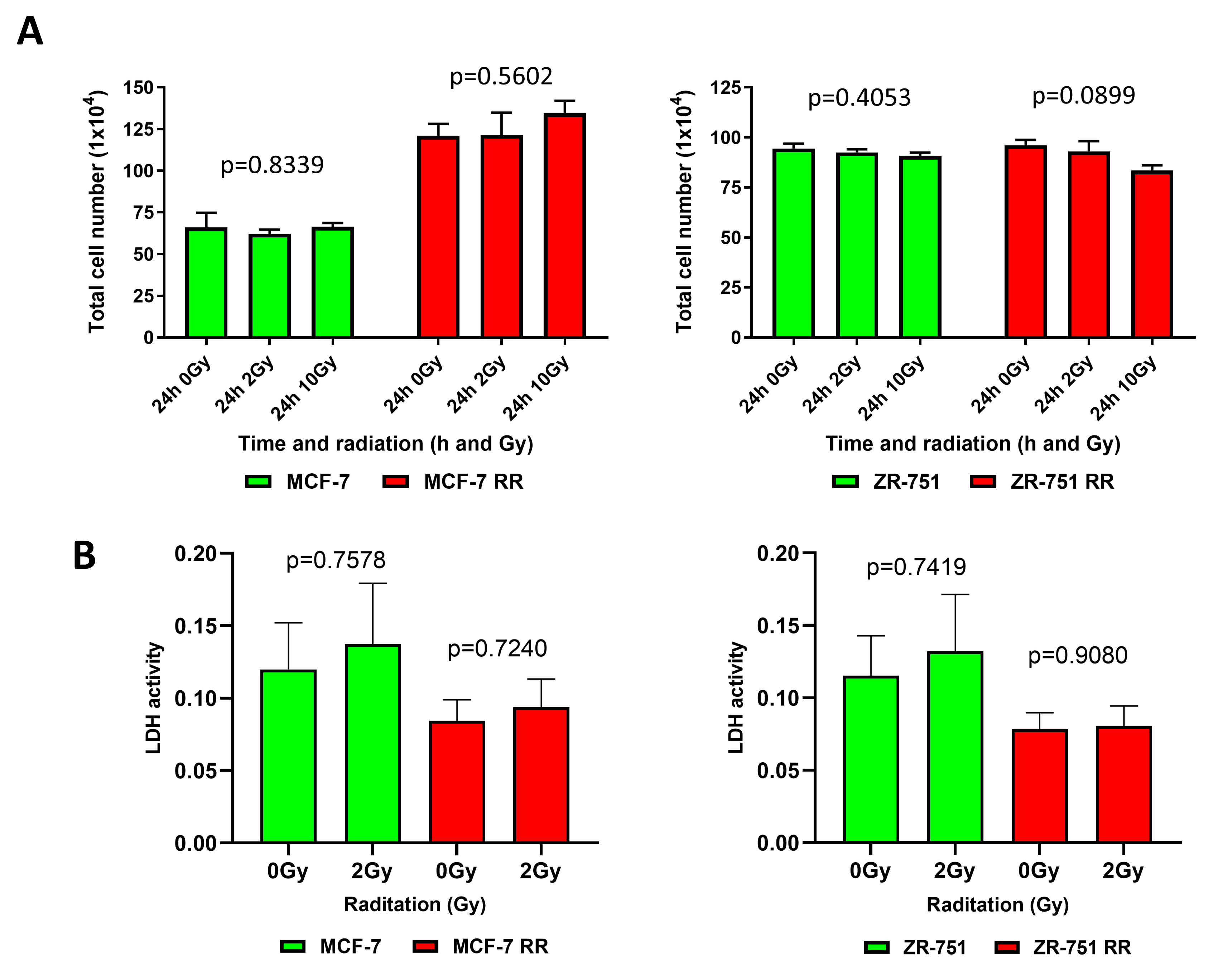

Supplement: Supplementary file 1 [file jpm-11-00796-s001.zip › Supplementary Figure S1.tif]

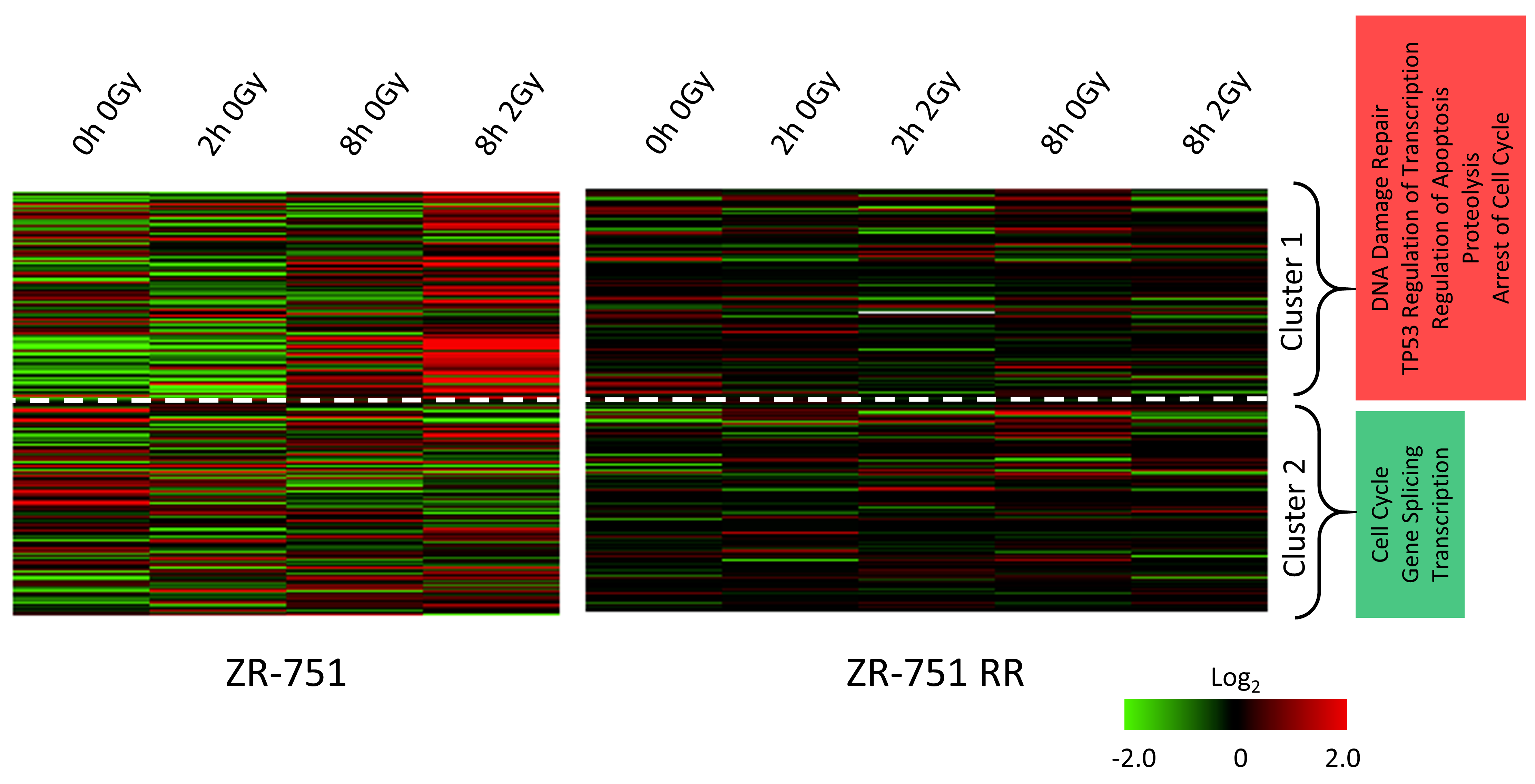

Supplement: Supplementary file 1 [file jpm-11-00796-s001.zip › Supplementary Figure S2.tif]

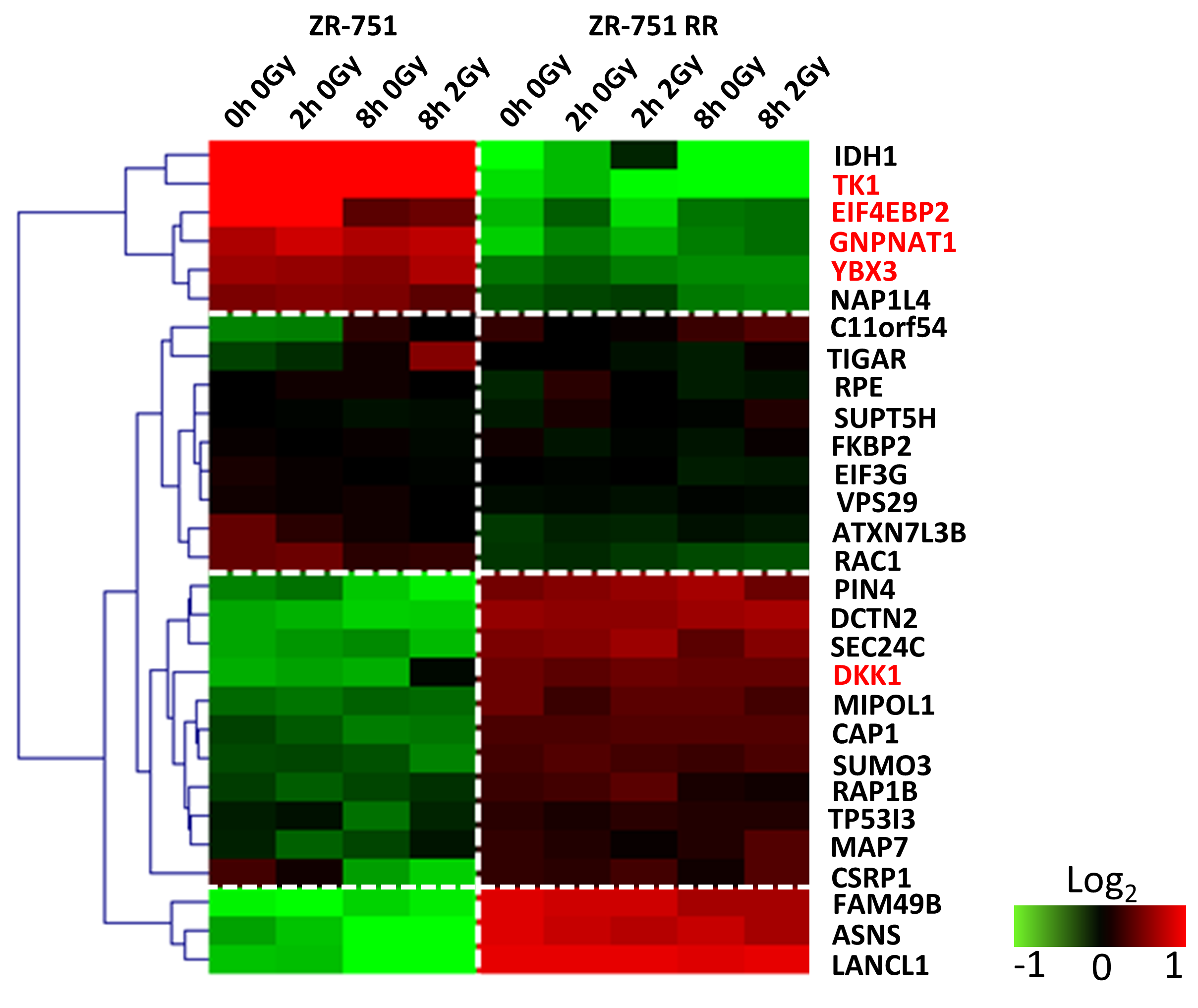

Supplement: Supplementary file 1 [file jpm-11-00796-s001.zip › Supplementary Figure S3.tif]

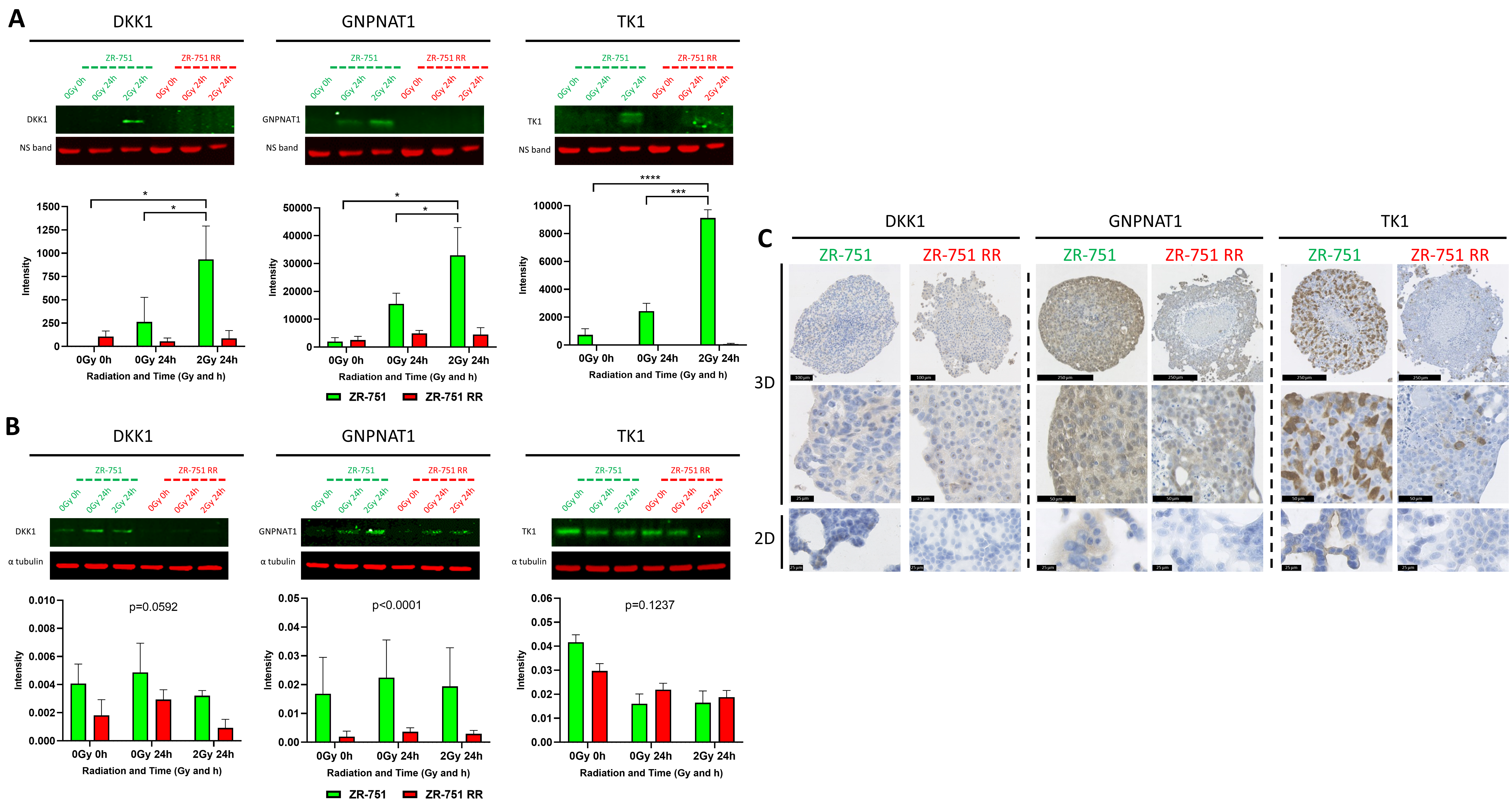

Supplement: Supplementary file 1 [file jpm-11-00796-s001.zip › Supplementary Figure S4.tif]

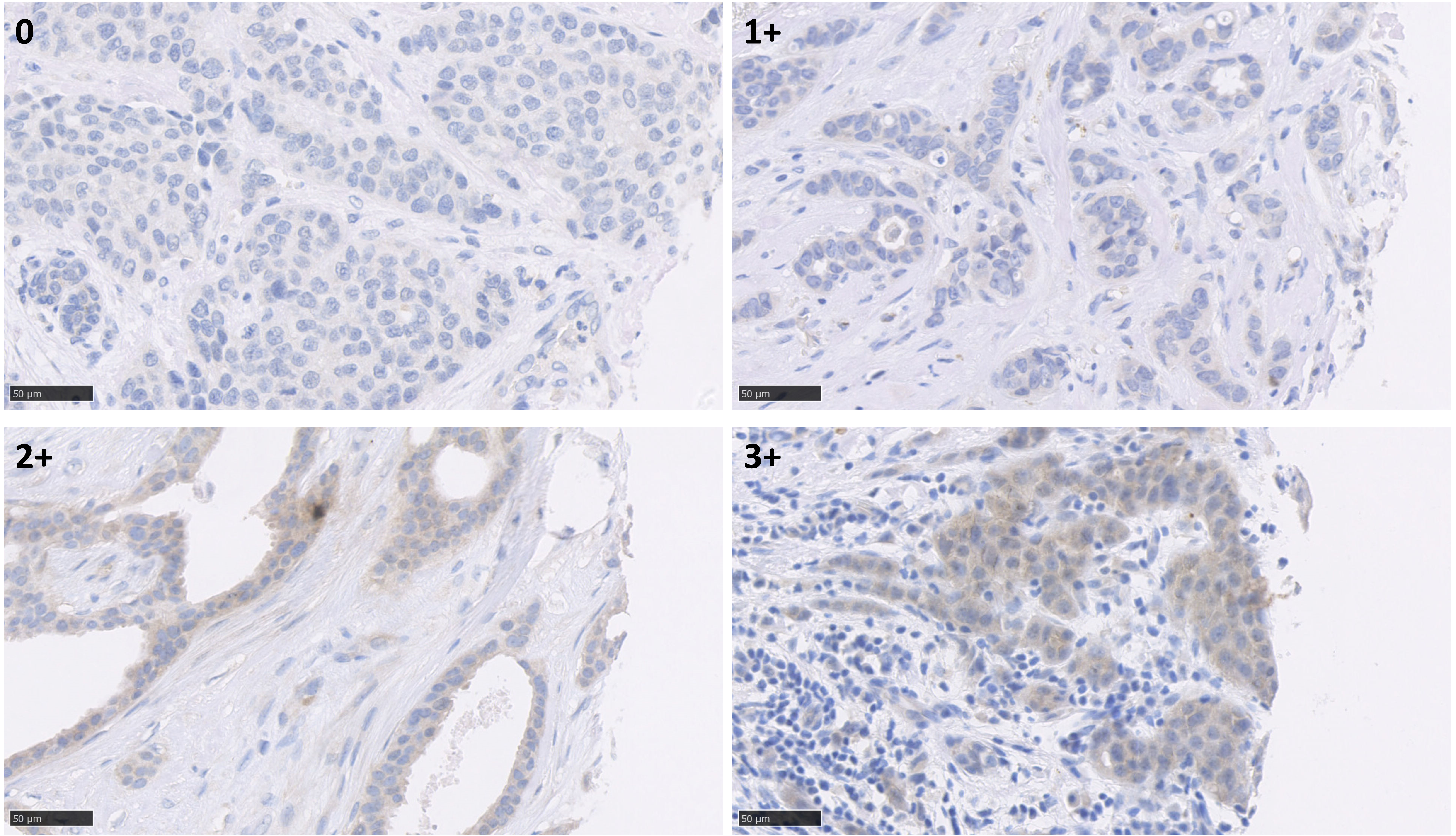

Supplement: Supplementary file 1 [file jpm-11-00796-s001.zip › Supplementary Figure S5.tif]

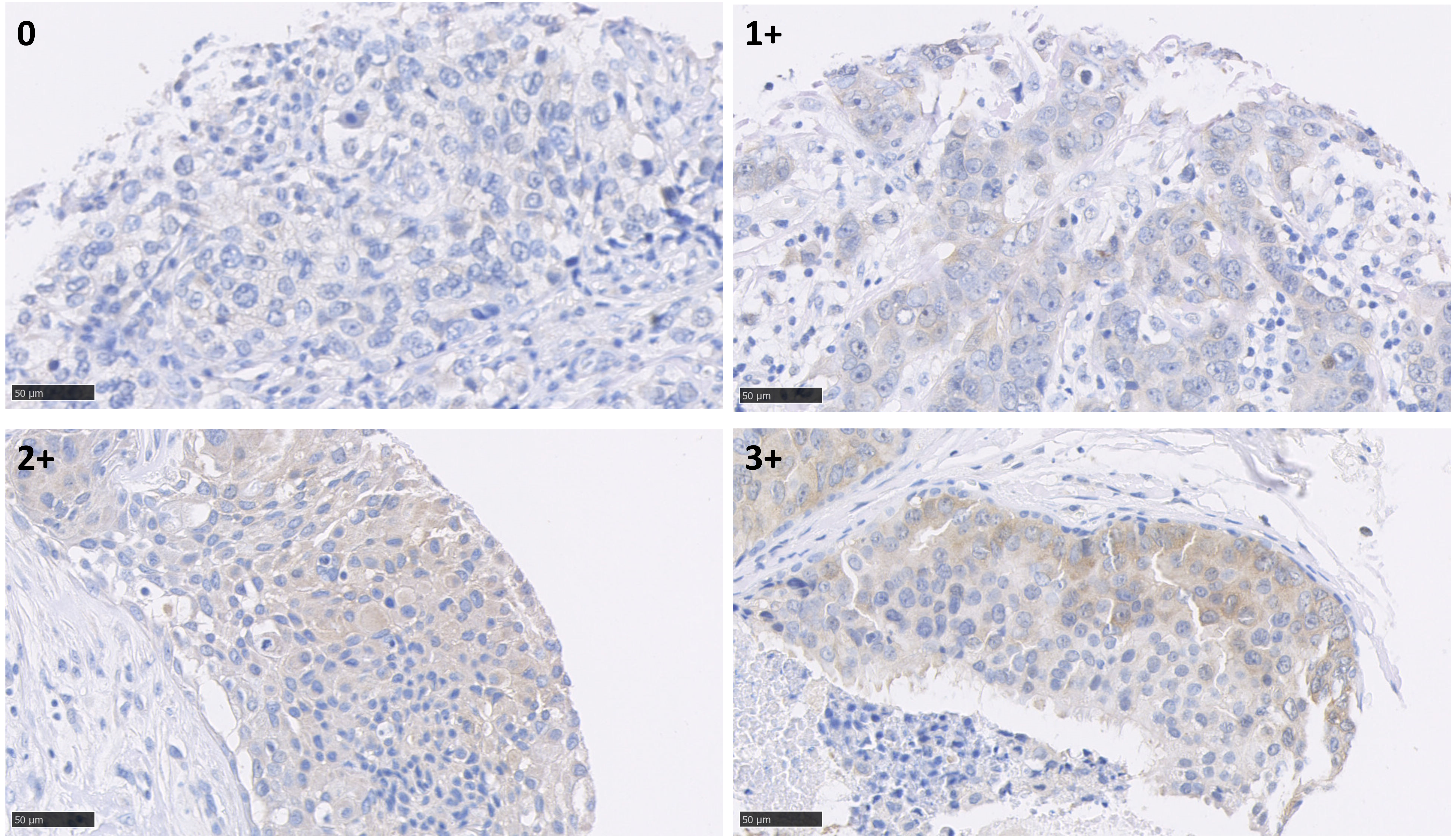

Supplement: Supplementary file 1 [file jpm-11-00796-s001.zip › Supplementary Figure S6.tif]

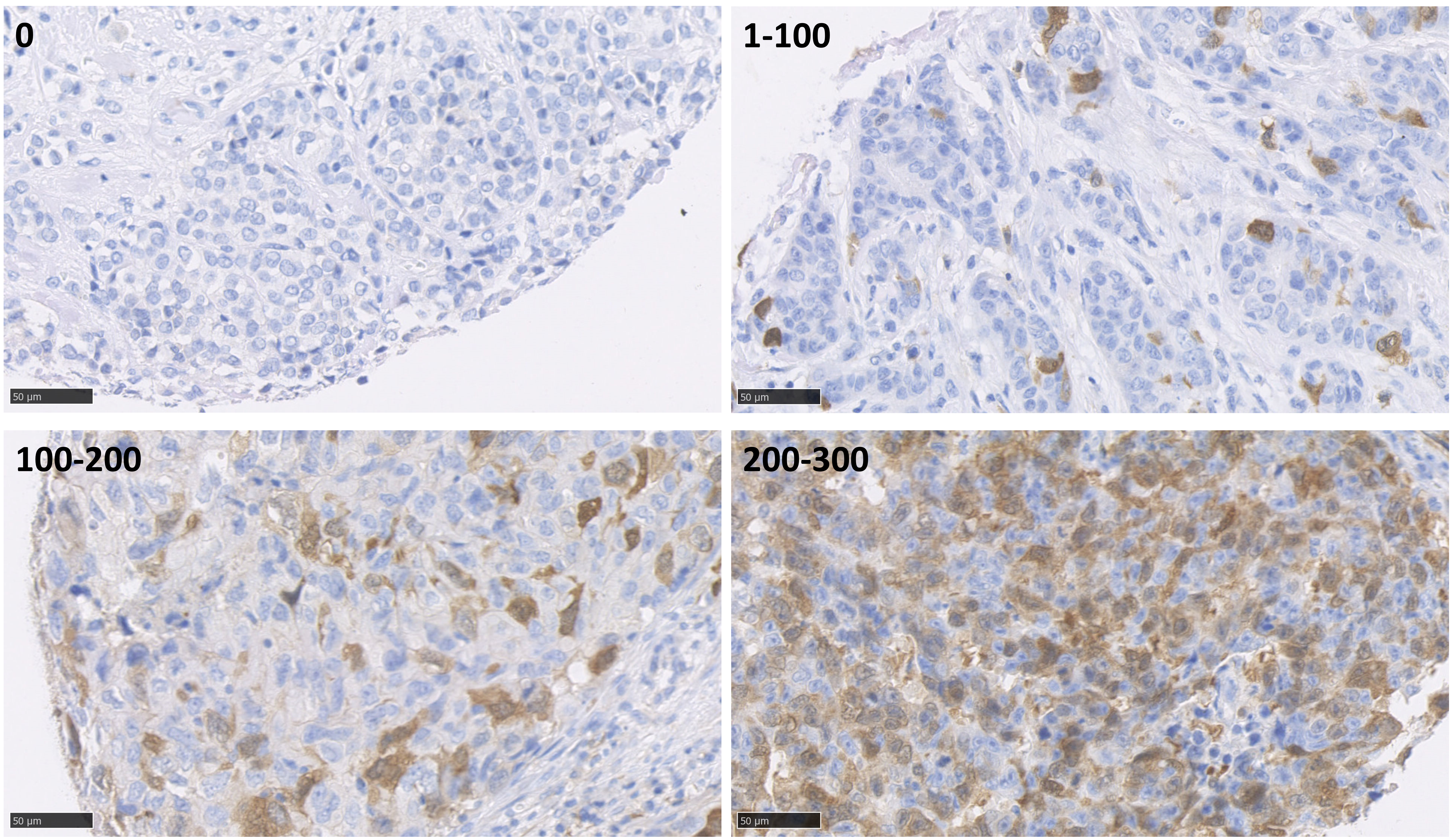

Supplement: Supplementary file 1 [file jpm-11-00796-s001.zip › Supplementary Figure S7.tif]
